# Kidney and Survival Outcomes with Semaglutide by CKD Severity in the FLOW Trial

**DOI:** 10.2215/CJN.0000000974

**Published:** 2026-02-18

**Authors:** Katherine R. Tuttle, Johannes F.E. Mann, Manuel M. Mayrdorfer, Brian Rayner, Giuseppe Pugliese, Richard E. Pratley, Vlado Perkovic, Kenneth W. Mahaffey, Naoki Kashihara, Ole K. Jeppesen, Janusz Gumprecht, Ricardo Correa-Rotter, David Z.I. Cherney, Heidrun Bosch-Traberg, Mustafa Arici, Peter Rossing

**Affiliations:** 1 Division of Nephrology, University of Washington School of Medicine, Seattle, Washington; 2 Providence Medical Research Center, Providence Inland Northwest Health, Spokane, Washington; 3 KfH Kidney Centre, München, Germany; 4 University Hospital, Friedrich-Alexander University, Erlangen, Germany; 5 Novo Nordisk A/S, Bagsværd, Denmark; 6 Groote Schuur Hospital, University of Cape Town, Cape Town, South Africa; 7 University of Rome La Sapienza, Rome, Italy; 8 AdventHealth Translational Research Institute, Orlando, Florida; 9 University of New South Wales, Sydney, New South Wales, Australia; 10 Department of Medicine, Stanford Center for Clinical Research (SCCR), Stanford, California; 11 Kawasaki Ika Daigaku, Kurashiki, Okayama, Japan; 12 Medical University of Silesia, Katowice, Poland; 13 Instituto Nacional de Ciencias Médicas y Nutrición Salvador Zubirán, Mexico City, Mexico; 14 Toronto General Hospital, Toronto, Ontario, Canada; 15 University of Toronto, Toronto, Ontario, Canada; 16 Hacettepe University Faculty of Medicine, Ankara, Turkey; 17 Steno Diabetes Center Copenhagen, Herlev, Denmark; 18 Kobenhavns Universitet, Kobenhavn, Denmark

**Keywords:** CKD, clinical trial, diabetes, diabetic nephropathy, diabetic kidney disease, GLP-1 receptor agonists

## Abstract

**Key Points:**

- Semaglutide reduced kidney outcome risk across broad CKD severity strata (eGFR; albuminuria).
- Reduced risk for all-cause death also carried through strata of CKD severity.
- In a population with type 2 diabetes, semaglutide improved kidney and survival outcomes irrespective of CKD severity, including advanced CKD.

**Background:**

Semaglutide improved kidney and overall survival in participants with type 2 diabetes (T2D) and CKD in the Evaluate Renal Function with Semaglutide Once Weekly (FLOW) trial. The aim of the present analyses was to quantify these benefits across broad strata of CKD severity.

**Methods:**

FLOW was a double-blind, randomized, placebo-controlled trial (median follow-up 3.4 [interquartile range, 2.9–4.0] years). Participants with T2D and eGFR 50–75 ml/min per 1.73 m^2^ and urine albumin-to-creatinine ratio (UACR) >300 to <5000 mg/g, or eGFR 25 to <50 ml/min per 1.73 m^2^ and UACR >100 to <5000 mg/g, were randomized to subcutaneous semaglutide 1 mg once-weekly or placebo. Subgroups were categorized by baseline eGFR (<30 to ≥60 ml/min per 1.73 m^2^) or UACR (<100 to ≥2000 mg/g) to assess the primary outcome and individual components (≥50% eGFR decline, eGFR <15 ml/min per 1.73 m^2^, dialysis, kidney transplant, and death due to kidney or cardiovascular causes), all-cause death, eGFR, and UACR.

**Results:**

At baseline, the mean±SD eGFR was 47±15 ml/min per 1.73 m^2^, and the median (5th–95th percentile) UACR was 568 (51–3225) mg/g. The primary outcome occurred in 19% (331 of 1767) versus 23% (410 of 1766) with semaglutide treatment versus placebo (hazard ratio [HR], 0.76; 95% confidence interval [CI], 0.66 to 0.88). Death occurred in 13% (227 of 1767) versus 16% (279 of 1766), respectively (HR, 0.80; 95% CI, 0.67 to 0.95). Across eGFR and UACR subgroups, HRs for the primary outcome remained consistent (*P* for interaction 0.83 and 0.42, respectively). For death, HRs were consistent among eGFR subgroups (*P* for interaction 0.54), but the HR was lowest (0.47; 95% CI, 0.31 to 0.70) for those with UACR ≥2000 mg/g (*P* for interaction 0.02). Estimated treatment effects on eGFR and UACR were generally consistent among subgroups.

**Conclusions:**

Semaglutide reduced risks of major kidney disease events and all-cause death across wide-ranging categories of baseline eGFR and UACR, supporting semaglutide treatment in T2D throughout the spectrum of CKD severity represented in FLOW, including advanced CKD.

Clinical Trial registry name and registration number: NCT03819153.

## Visual Abstract

**Figure:**
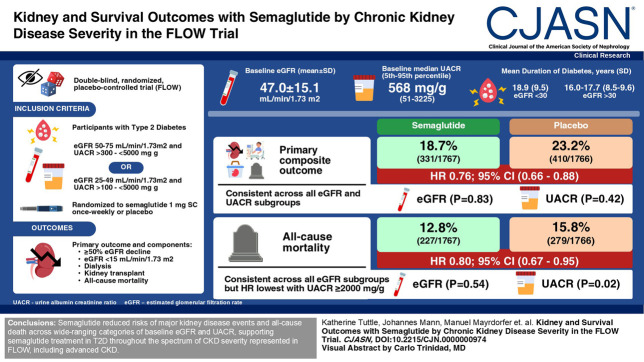

## Introduction

Diabetes has grown to pandemic proportions, with a global prevalence that is projected to become 783 million by 2045.^1^ Type 2 diabetes (T2D) is responsible for >90% of diabetes cases. Living with T2D leads to serious complications, including CKD in nearly four of ten people.^2,3^ As a result, T2D is now the most common cause of kidney failure worldwide.^4,5^ The Evaluate Renal Function with Semaglutide Once Weekly (FLOW) trial comparing subcutaneous semaglutide 1.0 mg once-weekly with placebo—the first dedicated kidney outcomes trial of a glucagon-like peptide-1 (GLP-1) receptor agonist in T2D—showed a relative risk reduction for the primary kidney outcome of 24% over a median of 3.4 years.^6,7^ The relative risk of all-cause death was also reduced by 20%.

Whether the kidney protective effects of semaglutide vary by CKD severity is unclear. In cardiovascular outcomes trials, GLP-1 receptor agonists reduced albuminuria and slowed eGFR decline, even when used with other potentially protective therapies in populations with T2D or obesity and relatively low CKD risk.^8–10^ At the other end of the spectrum, preservation of eGFR and albuminuria lowering was observed in a population with moderate-to-severe CKD treated by dulaglutide versus insulin glargine for T2D.^11^ Moreover, in the Trial to Evaluate Cardiovascular and Other Long-term Outcomes with Semaglutide in Subjects with Type 2 Diabetes and Peptide Innovation for Early Diabetes Treatment trials, slower eGFR decline or Kidney Disease Improving Global Outcomes risk improvement was most evident in lower eGFR subgroups, pointing to kidney protection with advancing CKD.^12^ Since death rates also escalate with eGFR decline,^13–19^ the aim of the present analyses was to quantify both kidney and survival benefits of semaglutide across discrete categories of eGFR and albuminuria representing the range of CKD severity in the FLOW trial.

## Methods

### Study Design and Participants

FLOW (ClinicalTrials.gov; NCT03819153) was a multicenter, double-blind, randomized (stratified according to the use of sodium-glucose cotransporter 2 [SGLT2] inhibitors at baseline), placebo-controlled clinical trial led by an academic steering committee and conducted at 387 international sites, with a median follow-up of 3.4 (interquartile range 2.9–4.0) years.^6,7^ Detailed inclusion and exclusion criteria have been published previously and are presented in Supplemental Table 1.^6,7^ The trial protocol and statistical analysis plan are in the Supplement. Adult participants (18 years or older) with T2D and CKD (baseline eGFR 50–75 ml/min per 1.73 m^2^ and urine albumin-to-creatinine ratio [UACR] >300 to <5000 mg/g, or eGFR 25 to <50 ml/min per 1.73 m^2^ and UACR >100 to <5000 mg/g) were randomized (1:1) to treatment with subcutaneous semaglutide 1.0 mg once-weekly or placebo. Participants received study treatment on top of standard of care including an angiotensin-converting enzyme inhibitor or an angiotensin II receptor blocker. The duration of FLOW was driven by the number of primary outcome events. Following an interim analysis, the Independent Data Monitoring Committee recommended early trial cessation when 570 events had occurred, because of overwhelming benefits.

### Outcomes

The primary outcome was a composite of ≥50% eGFR decline from baseline, kidney failure (onset of persistent eGFR <15 ml/min per 1.73 m^2^, initiation of chronic KRT by dialysis or kidney transplantation), and kidney or cardiovascular death.^6^ As prespecified secondary analyses, additional subgroups and outcomes are reported here: (*1*) The primary outcome by time-to-event analyses according to baseline eGFR (≥60, ≥45 to <60, ≥30 to <45, and <30 ml/min per 1.73 m^2^) and more granular UACR (<100, ≥100 to <300, ≥300 to <1000, ≥1000 to <2000, ≥2000 mg/g) subgroups; (*2*) individual components of the primary outcome by these eGFR and UACR subgroups; (*3*) estimated treatment effects on eGFR (both creatinine and cystatin-C based) and UACR change over time by these eGFR and UACR subgroups, respectively; and (*4*) all-cause death was similarly assessed by these eGFR and UACR subgroups.

Creatinine-based eGFR and cystatin C–based eGFR were measured in a central laboratory and calculated using the CKD Epidemiology Collaboration 2009 formula.^20^ UACR was also measured in a central laboratory and analyzed using spot urine samples.

### Statistical Analysis

FLOW was designed for 90% power to detect a 20% relative risk reduction for the primary kidney outcome. There were no power estimates for the subgroup analyses by baseline eGFR and UACR categories.

Aalen-Johansen estimates analyzed the time from randomization to the first primary outcome event, with noncardiovascular and nonkidney death modeled as competing risks. A Cox proportional hazards model (with treatment group and interaction between treatment group and subgroup as a fixed factor and stratified by use of SGLT2 inhibitors at baseline) evaluated treatment effects (hazard ratios [HRs] and 95% confidence intervals [95% CIs]) for the time from randomization to the first primary outcome event; participants without a first primary outcome event were censored at the end of their in-trial period. Events not defined by eGFR or UACR were confirmed by the Event Adjudication Committee.

Changes in baseline eGFR-creatinine, eGFR-cystatin C, and UACR over time were analyzed using a linear random effects model with treatment, use of SGLT2 inhibitors (yes/no) at baseline, time (as a continuous variable), and treatment time interaction as fixed effects, and including participant effect as a random intercept and time as a random slope. UACR at 24 months was also analyzed using an analysis of covariance model with treatment, use of SGLT2 inhibitors (yes/no) at baseline, baseline UACR subgroup, and interaction between treatment and baseline UACR subgroup as fixed factors and baseline UACR as a covariate. The ratio to baseline and the corresponding baseline value were log-transformed before analysis. Missing data at 24 months were imputed using a multiple imputation model and done separately for each treatment group and included baseline value as a covariate and use of SGLT-2 inhibitor (yes/no) at baseline as a fixed factor and was fitted to all subjects with a measurement regardless of treatment status at 24 months. The fitted model was used to impute values for all patients with missing data at 24 months to create 500 complete data sets. Rubin’s rules were used to combine the results.^21^

Time from randomization to all-cause death was analyzed using a Cox proportional hazards model with treatment as a categorical fixed factor and stratified by SGLT2 inhibitor use (yes versus no) at baseline. Participants without all-cause death were censored at the end of their in-trial period.

For subgroup analyses, estimated HRs and corresponding 95% CIs were calculated in a Cox proportional hazards model with treatment, subgroup, and interaction between treatment group and subgroup as fixed factors and stratified by SGLT2 inhibitor use (yes/no) at baseline. No adjustment for multiplicity or alpha-protection was performed and should therefore not be used to infer conclusive treatment effects. Two-sided *P* values <0.05 were considered significant. All statistical analyses were performed with SAS software, version 9.4 TS1M5 (SAS Institute).

## Results

### Participant Characteristics

Participant flow through the trial is shown in Supplemental Figure 1, as reported previously.^6^ Among 3533 participants in the FLOW population, 30% (*n*=1069) were women, mean (SD) age was 67 (9) years, mean (SD) eGFR was 47 (15) ml/min per 1.73 m^2^, and median (5th–95th percentile) UACR was 568 (51–3225) mg/g. Baseline characteristics were mostly balanced across eGFR subgroups (Table 1) with a few imbalances. The mean duration of diabetes in participants with eGFR <30 ml/min per 1.73 m^2^ was longer (18.9 [SD 9.5] versus 16.0–17.7 [8.5–9.6] years), and the use of insulin was higher (67% versus 56%–65% of participants) in contrast to other eGFR subgroups, respectively. Conversely, participants with baseline eGFR ≥45 ml/min per 1.73 m^2^ more frequently used angiotensin-converting enzyme inhibitors or angiotensin II receptor blockers (97%–98% versus 91%–94%, respectively) and SGLT2 inhibitors (19%–21% versus 11%–12%, respectively) than those with baseline eGFR <45 ml/min per 1.73 m^2^. Similarly, baseline characteristics were mostly balanced across UACR subgroups (Table 1). With higher UACR levels, the proportion of participants using insulin was higher.

**Table 1:** Baseline characteristics by baseline eGFR and urine albumin-to-creatinine ratio subgroups

Variable	Total (*N*=3533)^a^	eGFR Subgroup (ml/min per 1.73 m^2^)			
		≥60 (*n*=719)	≥45 to <60 (*n*=1055)	≥30 to <45 (*n*=1358)	<30 (*n*=400)
Age, yr	67±9	65±9	67±9	67±9	67±9
Female sex	1069 (30)	212 (29)	320 (30)	412 (30)	125 (31)
**History of CVD**					
ASCVD^b^	1198 (34)	239 (33)	385 (36)	446 (33)	128 (32)
Heart failure	678 (19)	118 (16)	227 (22)	258 (19)	75 (19)
HbA_1c_, %	7.8±1.3	7.9±1.3	7.8±1.3	7.8±1.3	7.6±1.3
Duration of diabetes, yr	17.4±9.3	16.0±8.5	17.2±9.2	17.7±9.6	18.9±9.5
eGFR, ml/min per 1.73 m^2^	47±15	70±9	51±4	37±4	26±2
UACR, mg/g	568 (51 to 3225)	562 (49 to 2839)	520 (43 to 3143)	567 (51 to 3414)	848 (78 to 3320)
ACE inhibitor/ARB use	3364 (95)	704 (98)	1023 (97)	1274 (94)	362 (91)
SGLT2 inhibitor use	550 (16)	151 (21)	198 (19)	158 (12)	43 (11)
Insulin use	2168 (61)	402 (56)	609 (58)	888 (65)	268 (67)

	Total (*N*=3533^a^)	UACR subgroup (mg/g)				
		<100 (*n*=350)	≥100 to <300 (*n*=763)	≥300 to <1000 (*n*=1288)	≥1000 to <2000 (*n*=640)	≥2000 (*n*=491)
Age, yr	67±9	69±8	68±8	67±9	66±9	63±10
Female sex	1069 (30)	139 (40)	225 (29)	373 (29)	184 (29)	148 (30)
**History of CVD**						
ASCVD^b^	1198 (34)	104 (30)	267 (35)	446 (35)	215 (34)	166 (34)
Heart failure	678 (19)	81 (23)	134 (18)	250 (19)	119 (19)	94 (19)
HbA_1c_, %	7.8±1.3	7.8±1.4	7.7±1.2	7.7±1.3	7.9±1.3	7.9±1.3
Duration of diabetes, yr	17.4±9.3	17.4±10.0	17.8±9.8	17.2±9.1	17.6±9.1	16.9±8.7
eGFR, ml/min per 1.73 m^2^	47±15	47±14	47±15	48±15	47±16	43±15
UACR, mg/g	568 (51 to 3225)	50 (5 to 96)	187 (109 to 289)	564 (325 to 941)	1410 (1028 to 1928)	2897 (2067 to 5340)
ACE inhibitor/ARB use	3364 (95)	334 (95)	726 (95)	1233 (96)	610 (95)	460 (94)
SGLT2 inhibitor use	550 (16)	60 (17)	135 (18)	190 (15)	106 (17)	59 (12)
Insulin use	2168 (61)	199 (57)	473 (62)	771 (60)	395 (62)	329 (67)

Values are no. (%), mean±SD, or median (5th to 95th percentiles). ACE, angiotensin-converting enzyme; ARB, angiotensin II receptor blocker; ASCVD, atherosclerotic cardiovascular disease; CVD, cardiovascular disease; HbA_1c_, glycated hemoglobin; SGLT2, sodium-glucose cotransporter 2; UACR, urine albumin-to-creatinine ratio.

a One participant in the semaglutide arm did not have baseline eGFR data available.

b Myocardial infarction, stroke, and peripheral arterial disease.

### Primary Outcome and Individual Components by eGFR and UACR Subgroups

**Figure 1:**
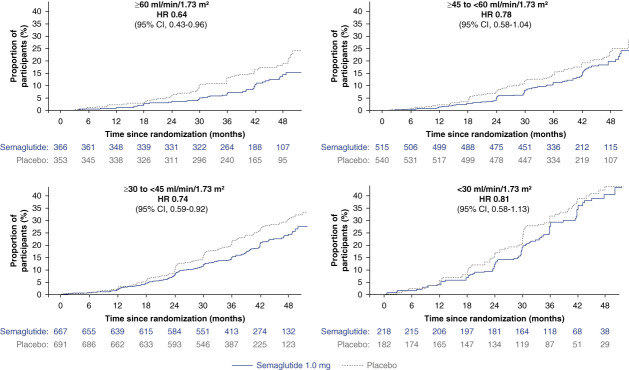
**Time to the primary kidney outcome by baseline eGFR subgroup.** *P* for interaction was 0.83. Cumulative incidence estimates based on time from randomization to first primary kidney outcome with noncardiovascular and nonkidney death modeled as competing risks during the in-trial period. CI, confidence interval; HR, hazard ratio.

The primary kidney outcome was consistent across eGFR subgroups, reported by 11% (41 of 366) versus 17% (59 of 353) of participants receiving semaglutide versus placebo, respectively, with eGFR ≥60 ml/min per 1.73 m^2^ (HR, 0.64; 95% CI, 0.43 to 0.96), 16% (80 of 515) versus 19% (103 of 540) with eGFR ≥45 to <60 ml/min per 1.73 m^2^ (HR, 0.78; 95% CI, 0.58 to 1.04), 21% (137 of 667) versus 26% (181 of 691) with eGFR≥30 to <45 ml/min per 1.73 m^2^ (HR, 0.74; 95% CI, 0.59 to 0.92), and 33% (73 of 218) versus 37% (67 of 182) with eGFR <30 ml/min per 1.73 m^2^ (HR, 0.81; 95% CI, 0.58 to 1.13; *P* value for interaction 0.83; Figure 1 and Supplemental Figure 2). Comparing semaglutide to placebo for the individual components^6^: eGFR decline of ≥50% was 9% (165 of 1766) versus 12% (213 of 1766) with a HR of 0.73 (95% CI, 0.59 to 0.89); sustained eGFR <15 ml/min per 1.73 m^2^ was 5% (92 of 1767) versus 6% (110 of 1766) with a HR of 0.80 (95% CI, 0.61 to 1.06); KRT occurred in 5% (87 of 1767) versus 6% (100 of 1766) with a HR of 0.84 (95% CI, 0.63 to 1.12); cardiovascular death occurred in 7% (123 of 1767) versus 10% (169 of 1766) with a HR of 0.71 (95% CI, 0.56 to 0.89). When the individual components were analyzed by eGFR subgroups, *P* values for interaction ranged between 0.28 and 0.86 (Supplemental Figure 2). The outcome of kidney death by eGFR subgroups could not be assessed because the number of events (ten cases) was too small.

**Figure 2:**
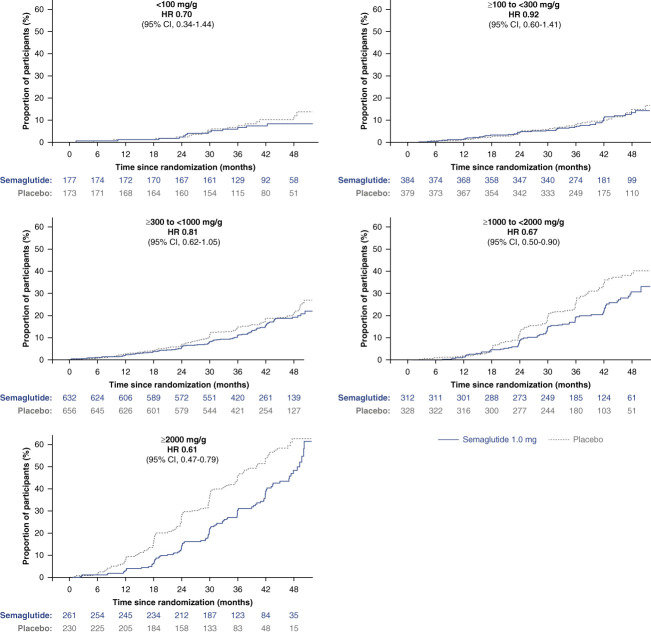
**Time to the primary kidney outcome by baseline UACR subgroup.** *P* for interaction was 0.42. Cumulative incidence estimates based on time from randomization to first primary kidney outcome with noncardiovascular and nonkidney death modeled as competing risks during the in-trial period. UACR, urine albumin-to-creatinine ratio.

Similarly consistent results for the primary kidney outcome were observed in the UACR subgroups (Figure 2 and Supplemental Figure 3), occurring in 7% (13 of 177) versus 10% (17 of 173) of participants receiving semaglutide versus placebo with UACR <100 mg/g (HR, 0.70; 95% CI, 0.34 to 1.44), 11% (42 of 384) versus 12% (45 of 379) with UACR ≥100 to <300 mg/g (HR, 0.92; 95% CI, 0.60 to 1.41), 15% (97 of 632) versus 18% (120 of 656) with UACR ≥300 to <1000 mg/g (HR, 0.81; 95% CI, 0.62 to 1.05), 24% (75 of 312) versus 33% (107 of 328) with UACR≥1000 to <2000 mg/g (HR, 0.67; 95% CI, 0.50 to 0.90), and 40% (104 of 261) versus 53% (121 of 230) with UACR ≥2000 mg/g (HR 0.61; 95% CI, 0.47 to 0.79; *P* value for interaction 0.42). When analyzed by UACR subgroups, the results were also consistent for eGFR decline of ≥50%, dialysis or kidney transplant, and cardiovascular death in the full analysis set, with *P* values for interaction ranging between 0.17 and 0.96 (Supplemental Figure 3). The outcomes of onset of persistent eGFR <15 ml/min per 1.73 m^2^ and kidney death could not be analyzed because of the low number of events in one or more UACR subgroups.

### Changes in eGFR Over Time by eGFR Subgroups

**Figure 3:**
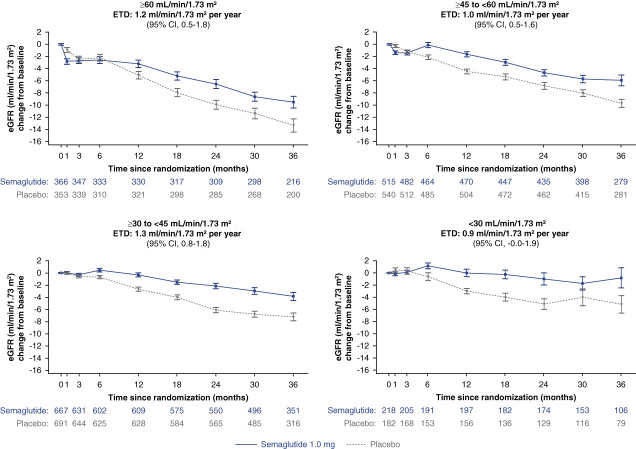
**Change in eGFR relative to baseline over time by eGFR subgroup.** *P* for interaction was 0.81. ETDs (ml/min per 1.73 m^2^ per year) between treatment with semaglutide and placebo are illustrated. Data are from the in-trial period. Error bars are ±SEM. Numbers shown in lower panels represent the number of participants contributing to the analysis at that time point. Change in eGFR was analyzed using a linear random effects model with treatment, use of SGLT2 inhibitors (yes/no) at baseline, time (as a continuous variable), and treatment time interaction as fixed effects, and including participant effect as a random intercept and time as a random slope. ETD, estimated treatment difference; SGLT2, sodium-glucose cotransporter 2.

The estimated treatment difference in mean creatinine-based eGFR at 24 months was 3.3 ml/min per 1.73 m^2^ (95% CI, 2.4 to 4.2) in the semaglutide versus placebo groups based on the full analysis set.^6^ Across eGFR subgroups, the rate of decline in eGFR was slower in participants receiving semaglutide than in those receiving placebo, with the following estimated treatment differences in mean eGFR slope (ml/min per 1.73 m^2^ per year): 1.2 (95% CI, 0.5 to 1.8) for eGFR≥60 ml/min per 1.73 m^2^, 1.0 (95% CI, 0.5 to 1.6) for ≥45 to <60 ml/min per 1.73 m^2^, 1.3 (95% CI, 0.8 to 1.8) for ≥30 to <45 ml/min per 1.73 m^2^, and 0.9 (95% CI, −0.0 to 1.9) for <30 ml/min per 1.73 m^2^ (interaction *P* = 0.81; Figure 3). Consistent benefits for semaglutide were also observed when evaluating rate of change in cystatin C–based eGFR (Supplemental Figure 4), with the estimated treatment differences in mean eGFR ranging between 1.1 and 1.8 ml/min per 1.73 m^2^ per year across the eGFR subgroups (interaction *P* = 0.34).

### Changes in Albuminuria Over Time by UACR Subgroups

Participants with albuminuria in the two lowest categories at baseline (<100 mg/g and ≥100 to <300 mg/g) had smaller changes in UACR relative to baseline at 24 months with semaglutide treatment compared with placebo (UACR geometric means at 24 months for semaglutide were 242.0 and 279.0 mg/g, respectively, and for placebo they were 347.2 and 402.0 mg/g, respectively) but had estimated treatment ratios of 0.70 and 0.69 on a relative scale, respectively (Supplemental Figure 5). For those with higher baseline albuminuria categories (≥300 to <1000 mg/g, ≥1000 to <2000 mg/g, and ≥2000 mg/g), semaglutide similarly decreased UACR compared with placebo on a relative scale (estimated treatment ratios of 0.70, 0.65, and 0.62, respectively) but with larger reductions (UACR geometric means at 24 months for semaglutide were 287.7, 338.6, and 344.2 mg/g, respectively, and for placebo, they were versus 409.1, 517.3, and 552.3 mg/g, respectively). The *P* for interaction was 0.94 across UACR subgroups.

### All-Cause Death by eGFR and UACR Subgroups

**Figure 4:**
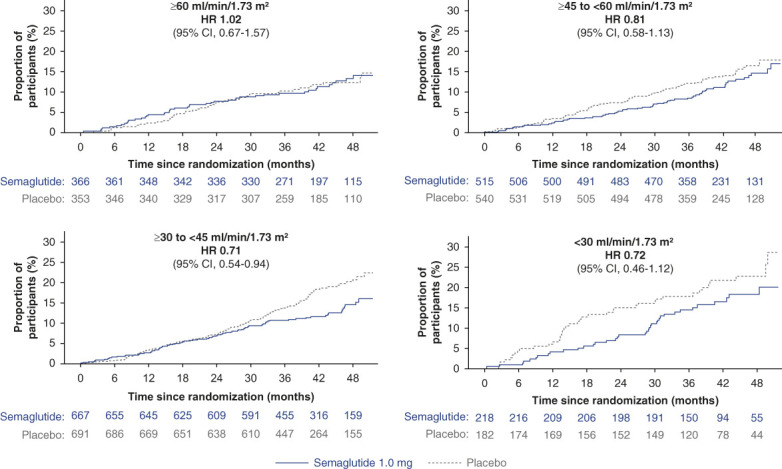
**Time to all-cause death by baseline eGFR subgroup.** *P* for interaction was 0.54. Time from randomization to all-cause death was analyzed using a Cox proportional hazards model with treatment as a categorical fixed factor and stratified by SGLT2 inhibitor use (yes/no) at baseline during the in-trial period.

All-cause death was a confirmatory secondary outcome in the FLOW trial.^6^ In eGFR subgroups, all-cause death occurred in 12% (44 of 366) versus 12% (42 of 353) of participants receiving semaglutide versus placebo with eGFR ≥60 ml/min per 1.73 m^2^ (HR, 1.02; 95% CI, 0.67 to 1.57), 12% (63 of 515) versus 15% (79 of 540) with eGFR ≥45 to <60 ml/min per 1.73 m^2^ (HR, 0.81; 95% CI, 0.58 to 1.13), 12% (83 of 667) versus 17% (118 of 691) with eGFR ≥30 to <45 ml/min per 1.73 m^2^ (HR, 0.71; 95% CI, 0.54 to 0.94), and 17% (37 of 218) versus 22% (40 of 182) with eGFR <30 ml/min per 1.73 m^2^ (HR, 0.72; 95% CI, 0.46 to 1.12; *P* for interaction 0.54; Figure 4 and Supplemental Table 2).

**Figure 5:**
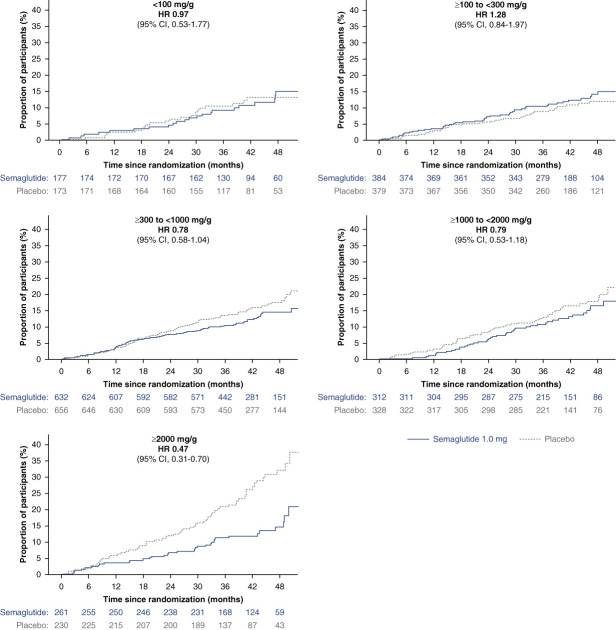
**Time to all-cause death by baseline UACR subgroup.** *P* for interaction was 0.02. Time from randomization to all-cause death was analyzed using a Cox proportional hazards model with treatment as a categorical fixed factor and stratified by SGLT2 inhibitor use (yes/no) at baseline during the in-trial period.

Across UACR subgroups, all-cause death outcome occurred in 12% (22 of 177) versus 12% (21 of 173) of participants receiving semaglutide versus placebo with UACR <100 mg/g (HR, 0.97; 95% CI, 0.53 to 1.77), 13% (48 of 384) versus 10% (38 of 379) with UACR ≥100 to <300 mg/g (HR, 1.28; 95% CI, 0.84 to 1.97), 12% (78 of 632) versus 16% (103 of 656) with UACR ≥300 to <1000 mg/g (HR, 0.78; 95% CI, 0.58 to 1.04), 14% (43 of 312) versus 17% (55 of 328) with UACR ≥1000 to <2000 mg/g (HR, 0.79; 95% CI, 0.53 to 1.18), and 14% (36 of 261) versus 27% (62 of 230) with UACR ≥2000 mg/g (HR, 0.47; 95% CI, 0.31 to 0.70) (Figure 5 and Supplemental Table 2). The *P* for interaction among UACR subgroups was 0.02, due to a greater benefit of semaglutide in the UACR ≥2000 mg/g subgroup versus the other UACR subgroups.

## Discussion

The FLOW trial enrolled a population with T2D across a range of CKD severity strata. Risk of the primary kidney outcome was consistently reduced throughout these strata determined by eGFR or UACR with semaglutide treatment versus placebo.^6^ Risks of primary outcome components inclusive of eGFR decline ≥50%, eGFR <15 ml/min per 1.73 m^2^, KRT by dialysis or transplant, or death due to cardiovascular causes were also consistently reduced by semaglutide in both eGFR and more granular UACR subgroups than previously reported. The evidence for kidney protection by semaglutide was further strengthened by findings of slower rates of eGFR decline (approximately 1 ml/min per 1.73 m^2^ per year or more compared with placebo) and reduced albuminuria (estimated treatment ratios of approximately 30%–40% compared with placebo) from the least to the most severe CKD subgroups. Moreover, time-to-event analyses revealed that benefits of semaglutide on the primary kidney outcome were evident early, at just 12–18 months, in the subgroup with UACR ≥2000 mg. In contrast to other subgroups, this UACR subgroup and the subgroup with eGFR<30 ml/min per 1.73 m^2^ appear to have similarly early overall survival benefits. Notably, participants with UACR ≥2000 mg had a >50% relative risk reduction for all-cause death.

Most FLOW participants (93%) had high-risk or very-high-risk CKD at baseline by the Kidney Disease Improving Global Outcomes classification.^6,22^ Lower eGFR and higher albuminuria independently and additively increase risks of cardiovascular and all-cause deaths.^16^ However, the risks of death and kidney failure in the diabetes population vary such that death is more common than kidney failure with eGFR >45 ml/min per 1.73 m^2^, but once eGFR is <30 ml/min per 1.73 m^2^, these trends reverse, although both rates remain exceedingly high.^19^ The present data support the use of semaglutide to preserve life, particularly in people with T2D and UACR >2000 mg/g, and perhaps in those with eGFR <30 ml/min per 1.73 m^2^. Indeed, such very-high-risk groups should be prioritized for implementation efforts, especially in the current scenario of high drug cost, limited access, and resource constraints.

The clinical benefits of semaglutide on CKD in T2D are likely to be multifactorial. Improvements in glycemic control, BP control, or weight loss may provide indirect effects to reduce these risks. However, in a mediation analysis for kidney outcomes in the Liraglutide Effect and Action in Diabetes: Evaluation of Cardiovascular Outcome Results and Semaglutide in Subjects with Type 2 Diabetes-6 trials, only 9% and 22% of risk reduction was attributable to lower BP or glycemia, respectively, with no relationship to body weight.^23^ In addition, no relationship between change in body weight and eGFR was observed in clinical trials of a conventional GLP-1 receptor agonist, dulaglutide, or a dual GLP-1 and gastrointestinal insulinotropic peptide agonist, tirzepatide, in study populations with T2D.^24,25^ Therefore, direct protective effects by GLP-1 receptor agonists acting through other mechanisms are likely.

GLP-1 receptors are expressed throughout the body.^26^ In the kidney, endothelial cells and vascular smooth muscle cells of the juxtaglomerular apparatus are the main location where GLP-1 receptors have been detected.^27^ As this is a relatively small cell population, nonintrinsic kidney cells could also be involved. Macrophages and *T* lymphocytes promote inflammation and fibrosis within organs throughout the body, including the kidney and heart.^28–30^ These immune cells contain GLP-1 receptors, that when activated, convert them to less or anti-inflammatory phenotypes.^28^ Mouse and rat models show that signals through proinflammatory receptors, such as the receptor for advanced glycation end products and Toll-like receptors, are suppressed by GLP-1 receptor agonists, possibly by central nervous system GLP-1 actions resulting in immunomodulation through parasympathetic and opioid efferent activity.^31–33^ Accordingly, GLP-1 receptor agonists reduce kidney inflammation and fibrosis in mouse and rat models of CKD with or without diabetes.^31,32^ In humans with T2D and CKD, dulaglutide correspondingly reduced profibrotic biomarkers in blood and urine.^34^ Interestingly, in a mouse model of sepsis, liraglutide decreased lung injury and reduced the death rate,^33^ suggesting that resistance to severe illness could be another potential mechanistic link to improved overall survival as well as fewer serious infections and coronavirus disease 2019–related adverse events in FLOW.^6^

The present analyses have strengths and limitations. We assessed the primary kidney outcome, other secondary kidney outcomes, and all-cause death in subgroups defined according to eGFR and UACR categories. Although these subgroups were prespecified, they were not powered for these outcomes, and some had wide CIs giving uncertainty to individual estimates. In addition, some were too small to assess, *i.e*., kidney death by either eGFR or UACR categories and eGFR <15 ml/min per 1.73 m^2^ for UACR categories. The all-cause death analyses by CKD severity were conducted *post hoc*, which limits interpretability to exploratory observations. Nevertheless, statistical heterogeneity was not observed for eGFR categories, and the greatest benefit on all-cause death was seen in the highest UACR subgroup. At a minimum, this high-risk group benefits at least as much as others, and the benefit appears early when death is a major risk. Finally, the FLOW population was comprised of mostly older people, men, and those who identified as White race. To better align with representation of T2D and CKD populations, studies of diverse groups are needed to more fully understand the kidney and survival benefits of semaglutide.

In conclusion, semaglutide meaningfully reduced risks of major kidney disease events and all-cause death across multiple strata of CKD severity, on top of the current standard of care, in the FLOW population with T2D, including those with low levels of eGFR or high levels of albuminuria. Semaglutide is an important therapeutic consideration for improving kidney and survival outcomes for people with T2D and CKD including those with advanced CKD.

## Supplementary Material

## Data Availability

Data belong to a third party, and authors are not authorized to share the data. Identity of Third Party: Novo Nordisk A/S. Reason for Restriction: An authorised researcher can request access to clinical study data by submitting a research proposal for review and approval by Novo Nordisk and an internal independent review panel. Requests are usually considered after the research is finished and the main results have been published. If the research supports a regulatory application, requests will be considered after the product and its intended use are approved in both the European Union and the United States of America. Participants clinical data will be anonymised, following an approved internal process, before data are shared to external third parties. For details on how to request access to clinical data, visit novonordisk-trials.com.

## Declarative Statements

This study includes clinical experimentation, and the FLOW trial was conducted in accordance with the Declaration of Helsinki and received Institutional Review Board or Ethics Committee approval. All patients provided written informed consent.

## Supplemental Material

Supplemental Table 1. Inclusion and exclusion criteria (as described previously^1,2^).

Supplemental Table 2. All-cause death by baseline eGFR and UACR subgroup.

Supplemental Figure 1. Participant flow through the FLOW trial.^1^

Supplemental Figure 2. The primary kidney outcome and its individual components by baseline eGFR subgroup.

Supplemental Figure 3. The primary kidney outcome and its individual components by baseline UACR subgroup.

Supplemental Figure 4. Change in eGFR (cystatin C) relative to baseline over time by eGFR subgroup.

Supplemental Figure 5. Change in UACR relative to baseline over time by UACR subgroup.

Supplemental References.

FLOW Trial Investigators List.
