# Supplementary material for: Kidney and Survival Outcomes with Semaglutide by CKD Severity in the FLOW Trial
Source: Clin J Am Soc Nephrol. 2026 Feb 18;21(5):841–51. doi: 10.2215/CJN.0000000974 (PMC13143484; doi:10.2215/CJN.0000000974)
Supplement: Supplementary file 1 [file cjasn-21-841-s001.pdf]

Supplemental Material

Kidney and Survival Outcomes with Semaglutide by Chronic Kidney Disease Severity in the FLOW Trial

Tuttle KR, et al.

Contents

Supplemental Table 1. Inclusion and exclusion criteria (as described previously[1,2]) 2

Supplemental Table 2. All-cause death by baseline eGFR and UACR subgroup 4

Supplemental Figure 1. Participant flow through the FLOW trial.[1] 5

Supplemental Figure 2. The primary kidney outcome and its individual components by baseline eGFR subgroup. ...6

Supplemental Figure 3. The primary kidney outcome and its individual components by baseline UACR subgroup. ..8

Supplemental Figure 4. Change in eGFR (cystatin C) relative to baseline over time by eGFR subgroup. 10

Supplemental Figure 5. Change in UACR relative to baseline over time by UACR subgroup. 12

FLOW trial investigators[1] 13

References 18

Supplemental Table 1. Inclusion and exclusion criteria (as described previously[1,2])

Inclusion criteria	Exclusion criteria
• Signed informed consent[a]<br>• Male or female<br>• Aged ≥18 years (≥20 years in Japan) at the time of signing informed consent<br>• Diagnosed with T2D<br>• HbA_{1c} ≤10% (≤86 mmol/mol)[b]<br>• Renal impairment defined by either:<br>o Serum creatinine–based eGFR between ≥50 and ≤75 mL/min/1.73 m^2 (CKD-EPI),[b,c] and UACR between >300 and <5000 mg/g[b]<br>Or<br>o Serum creatinine–based eGFR between ≥25 and <50 mL/min/1.73 m^2 (CKD-EPI),[b] and UACR between >100 and <5000 mg/g[b]<br>• Treatment with maximum labeled or tolerated dose of a RAAS-blocking agent, including an ACE inhibitor or an ARB (unless contraindicated or not tolerated[d])	• Known or suspected hypersensitivity to trial product(s) or related products<br>• Pregnancy, breastfeeding or intention to become pregnant, or of child-bearing potential and not using a highly effective contraceptive method<br>• Participation in any clinical trial of an approved or nonapproved investigational medicinal product within 30 days (1 year in Brazil) before screening[e]<br>• Any disorder that in the investigator's opinion might have jeopardized the patient's safety or compliance with the protocol<br>• Congenital or hereditary kidney diseases[f]<br>• Use of any GLP-1 receptor agonist within 30 days prior to screening<br>• Personal or first-degree relative(s) history of MEN2 or MTC<br>• Myocardial infarction, stroke, hospitalization for unstable angina pectoris, or transient ischemic attack within 60 days prior to screening<br>• Currently classed with New York Heart Association Class IV heart failure<br>• Planned coronary, carotid, or peripheral artery revascularization<br>• Chronic or intermittent hemodialysis or peritoneal dialysis within 90 days<br>• Uncontrolled and potentially unstable diabetic retinopathy or maculopathy[g]<br>• Presence or history of malignant neoplasm within 5 years prior to the day of screening[h]<br>• A prior solid organ transplantation, or awaiting a solid organ transplantation<br>• ACE inhibitor and ARB combination therapy

ACE, angiotensin-converting enzyme; ARB, angiotensin II receptor blocker; CKD-EPI, Chronic Kidney Disease Epidemiology Collaboration; eGFR, estimated glomerular filtration rate; GLP-1, glucagon-like peptide 1; HbA1c, glycated hemoglobin; MEN2, multiple endocrine neoplasia type 2; MTC, medullary thyroid carcinoma; RAAS, renin-angiotensin-aldosterone system; T2D, type 2 diabetes; UACR, urine albumin-to-creatinine ratio.

[a]Obtained before any trial-related activities (i.e., any procedures conducted as part of the trial, such as determining suitability for the trial, except for protocol-described prescreening activities that require separate informed consent).

[b]Laboratory results for inclusion were based on measurements no more than 90 days old at screening, documented in medical records, or measurements from the optional prescreening visit, documented in medical records, or central laboratory measurement obtained at the screening visit. The patient must have been in usual health condition at the time of sample collection used for inclusion as evaluated by the investigator and treated with a RAAS blocking agent.

[c]The number of patients with inclusion eGFR ≥60 mL/min/1.73 m^2 was capped at 20% of randomized patients.

[d]Treatment dose must have been stable for ≥4 weeks prior to the date of the laboratory assessments used for determination the renal impairment inclusion criterion, and kept stable until screening.

[e]Simultaneous participation in a trial with the primary objective of evaluating an approved or nonapproved investigational medicinal product for prevention or treatment of COVID-19 disease or post-infectious conditions was allowed if the last dose of the investigational medicinal product had been received more than 30 days before screening.

[f]Including polycystic kidney disease, autoimmune kidney diseases including glomerulonephritis, or congenital urinary tract malformations.

[g]Verified by fundus examination within 90 days prior to screening or in the period between screening and randomization. Pharmacological pupil dilation was a requirement, unless using a digital fundus photography camera specified for nondilated examination.

[h]Basal and squamous cell skin cancer and any carcinoma in situ were allowed.

Supplemental Table 2. All-cause death by baseline eGFR and UACR subgroup

	Semaglutide 1.0 mg<br>Events/analyzed participants	Placebo<br>Events/analyzed participants	HR (95% CI)	*P* for interaction
**eGFR subgroup (mL/min/1.73 m^2)**				
≥60	44/366	42/353	1.02 (0.67-1.57)	0.54
≥45 to <60	63/515	79/540	0.81 (0.58-1.13)	
≥30 to <45	83/667	118/691	0.71 (0.54-0.94)	
<30	37/218	40/182	0.72 (0.46-1.12)	
**UACR subgroup (mg/g)**				
<100	22/177	21/173	0.97 (0.53-1.77)	0.02
≥100 to <300	48/384	38/379	1.28 (0.84-1.97)	
≥300 to <1000	78/632	103/656	0.78 (0.58-1.04)	
≥1000 to <2000	43/312	55/328	0.79 (0.53-1.18)	
≥2000	36/261	62/230	0.47 (0.31-0.70)	

Data from the in-trial period. Time from randomization to all-cause death was analyzed using a Cox proportional hazards model with treatment as a categorical fixed factor and stratified by SGLT2 inhibitor use (yes/no) at baseline during the in-trial period. CI, confidence interval; HR, hazard ratio; SGLT2, sodium–glucose cotransporter-2; UACR, urine albumin-to-creatinine ratio.

Supplemental Figure 1. Participant flow through the FLOW trial.[1]

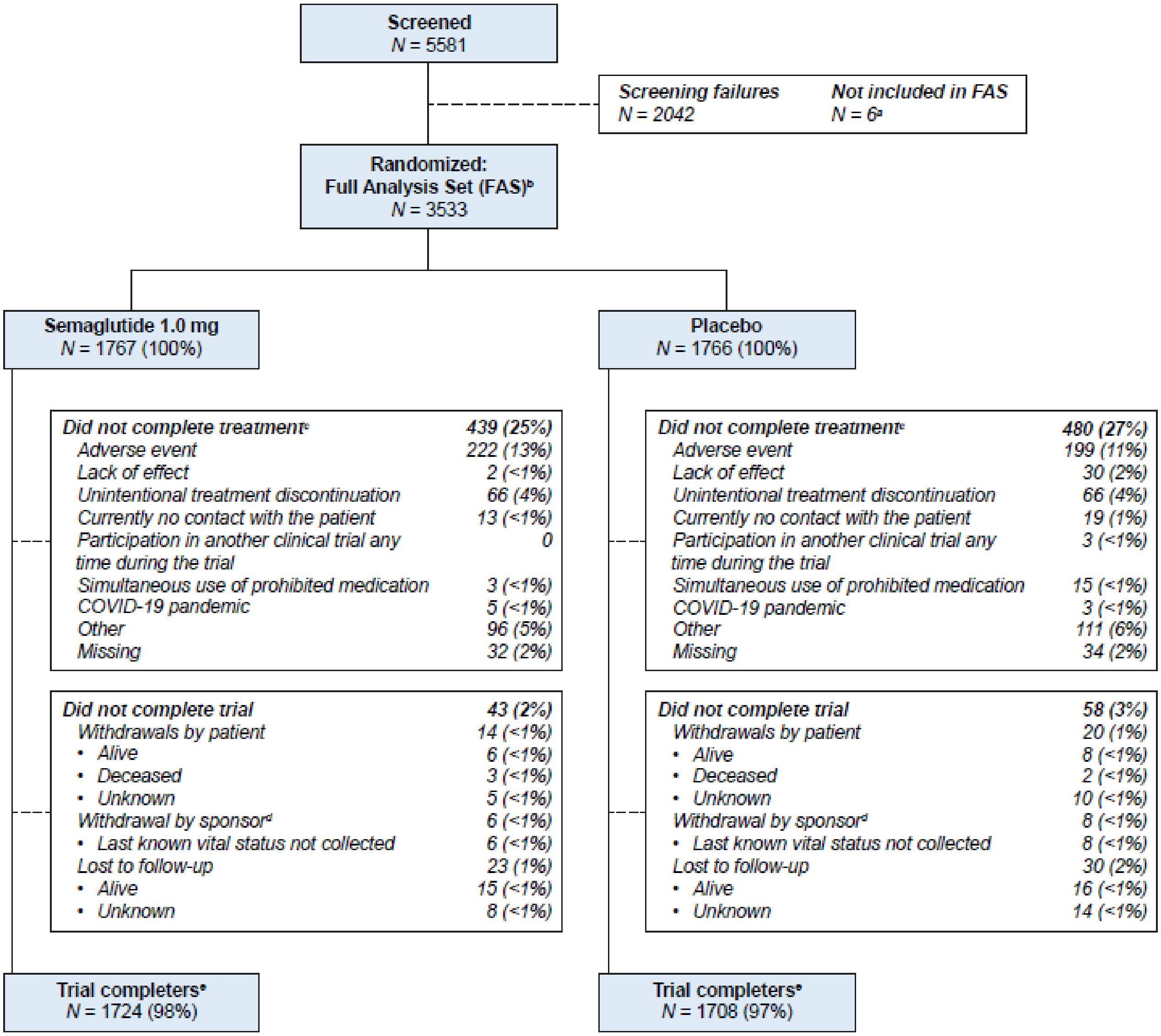

[a]Five participants were randomized more than once, and 1 participant was excluded due to good clinical practice issues at site. [b]All participants in the FAS were included in the analyses of primary and confirmatory secondary endpoints. [c]Permanent treatment discontinuation more than 30 days prior to the end-of-treatment visit. [d]Following the closure of 2 sanctioned sites in Russia. [e]Patients who attended the follow-up visit or died during the trial.

COVID-19, coronavirus disease 2019; FAS, full analysis set.

Supplemental Figure 2. The primary kidney outcome and its individual components by baseline eGFR subgroup.

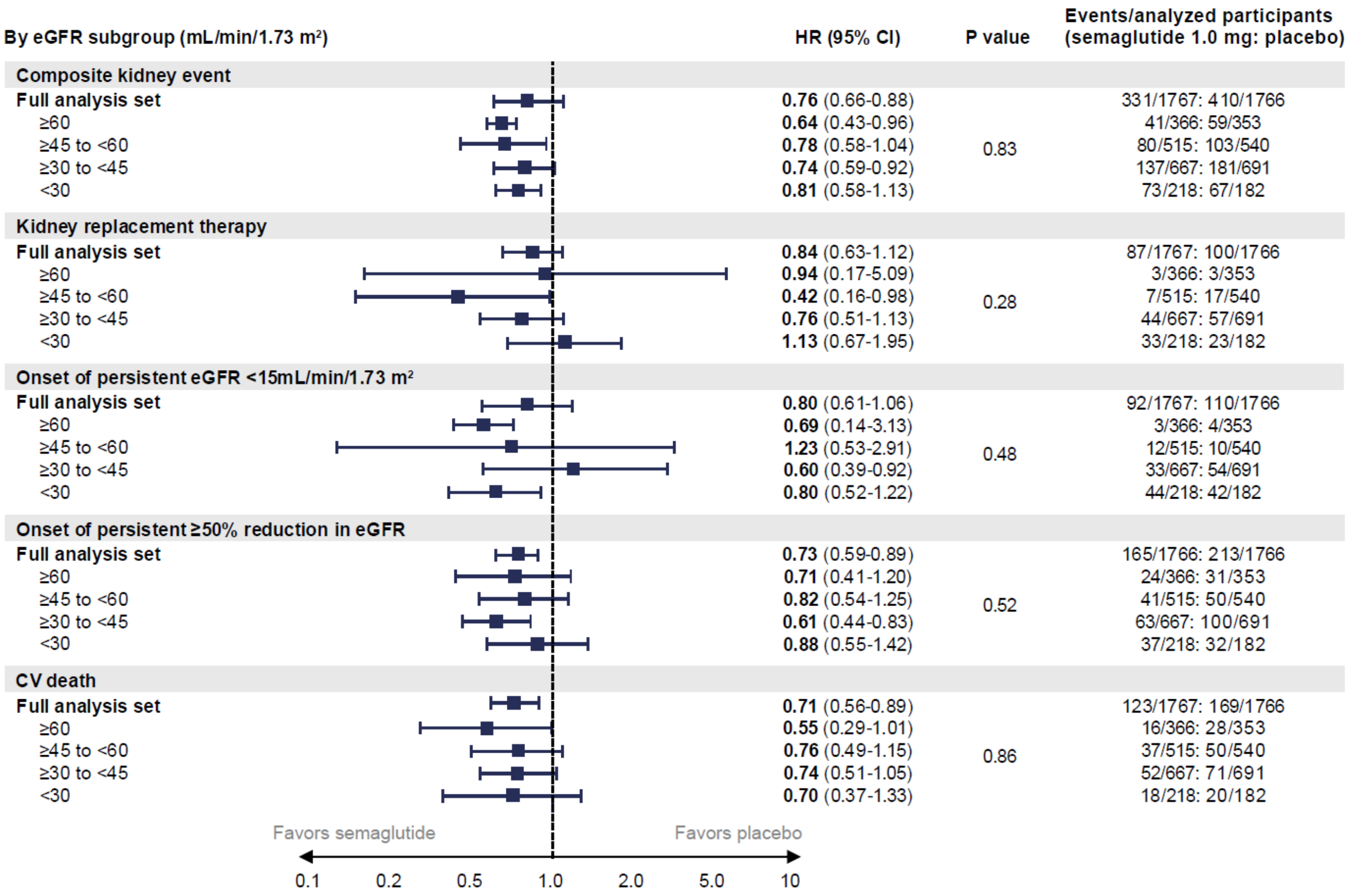

Data from the in-trial period. Time from randomization to the primary kidney outcome was analyzed using a Cox proportional hazards model with treatment as a categorical fixed factor and stratified by the use of SGLT2 inhibitors (yes/no) at baseline. Kidney replacement therapy comprised dialysis or kidney

transplantation. There were too few kidney deaths to perform the subgroup analysis. *P* values for interaction are shown. CI, confidence interval; CV, cardiovascular; eGFR, estimated glomerular filtration rate; HR, hazard ratio; SGLT2, sodium–glucose cotransporter-2.

Supplemental Figure 3. The primary kidney outcome and its individual components by baseline UACR subgroup.

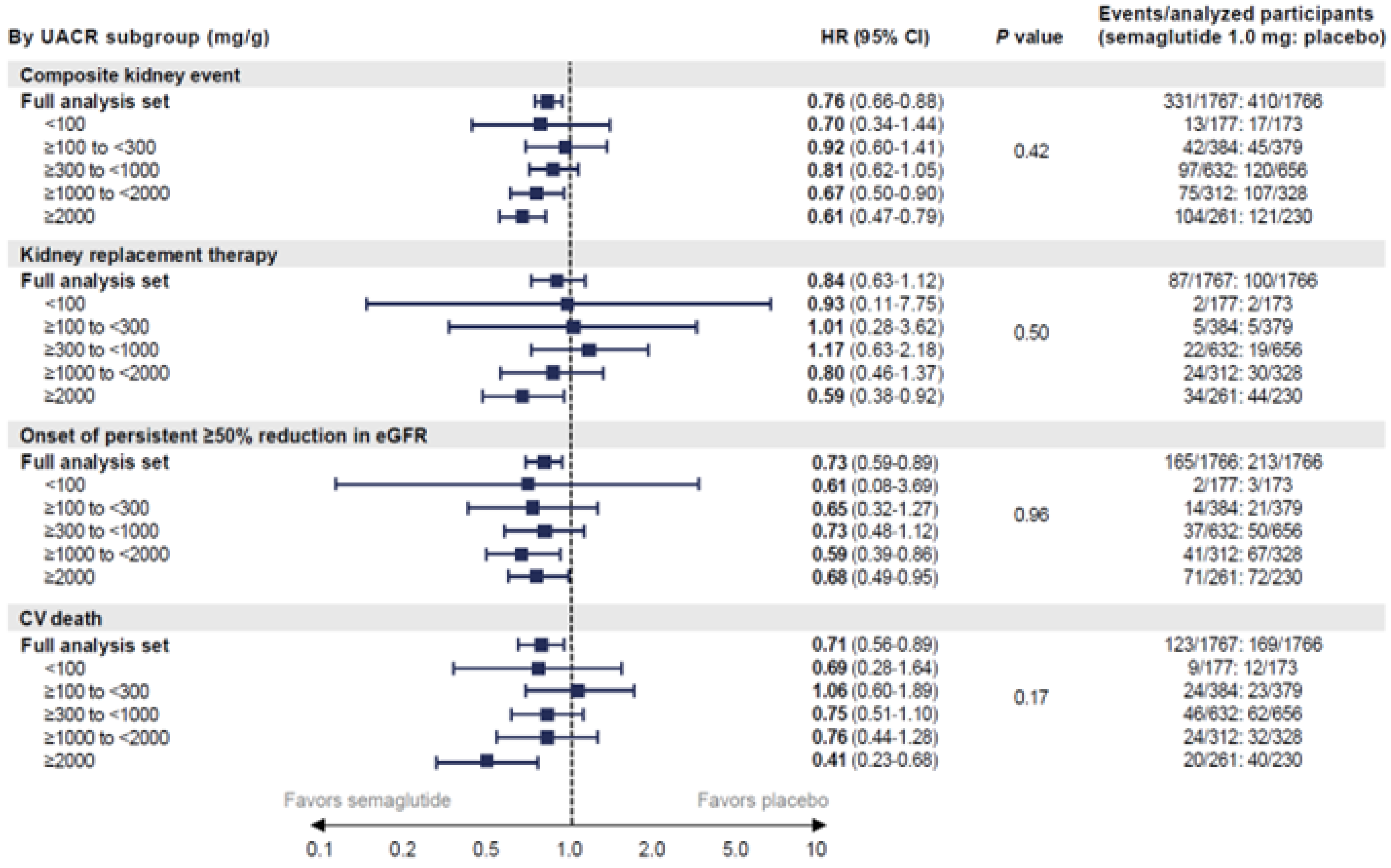

Data from the in-trial period. Time from randomization to the primary kidney outcome was analyzed using a Cox proportional hazards model with treatment as a categorical fixed factor and stratified by the use of SGLT2 inhibitors (yes/no) at baseline. Participants without events of interest were censored at the end of their in-trial period. Kidney replacement therapy comprised dialysis or kidney transplantation. There were too few events for onset of persistent eGFR <15 mL/min/1.73 m^2 and kidney death to perform

the subgroup analyses. *P* values for interaction are shown. CI, confidence interval; CV, cardiovascular; eGFR, estimated glomerular filtration rate; HR, hazard ratio; SGLT2, sodium–glucose cotransporter-2; UACR, urine albumin-to-creatinine ratio.

Supplemental Figure 4. Change in eGFR (cystatin C) relative to baseline over time by eGFR subgroup.

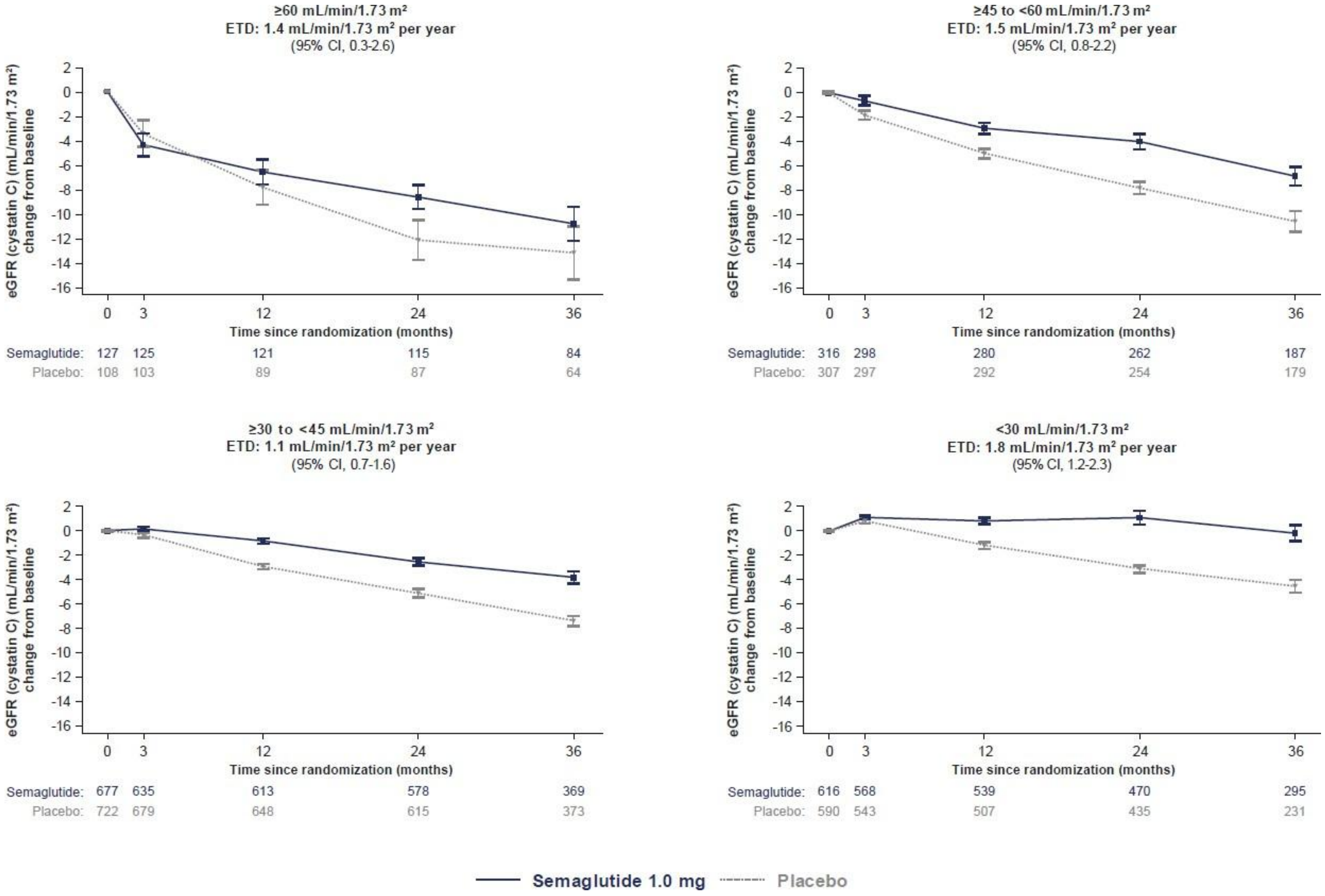

P for interaction was 0.34. ETDs (mL/min/1.73 m^2 per year) between treatment with semaglutide and placebo are illustrated. Data are from the in-trial period. Error bars are +/− standard error. Numbers shown in lower panels represent the number of participants contributing to the analysis at that timepoint. Change in eGFR was analyzed using a linear random effects model with treatment, use of SGLT2 inhibitors (yes/no) at baseline, time (as a continuous variable) and treatment time interaction as fixed effects, and including participant effect as a random intercept and time as a random slope. Cystatin C–eGFR was calculated using the CKD-EPI 2009 formula. CI, confidence interval; CKD-EPI, Chronic Kidney Disease Epidemiology Collaboration; eGFR, estimated glomerular filtration rate; ETD, estimated treatment difference.

Supplemental Figure 5. Change in UACR relative to baseline over time by UACR subgroup.

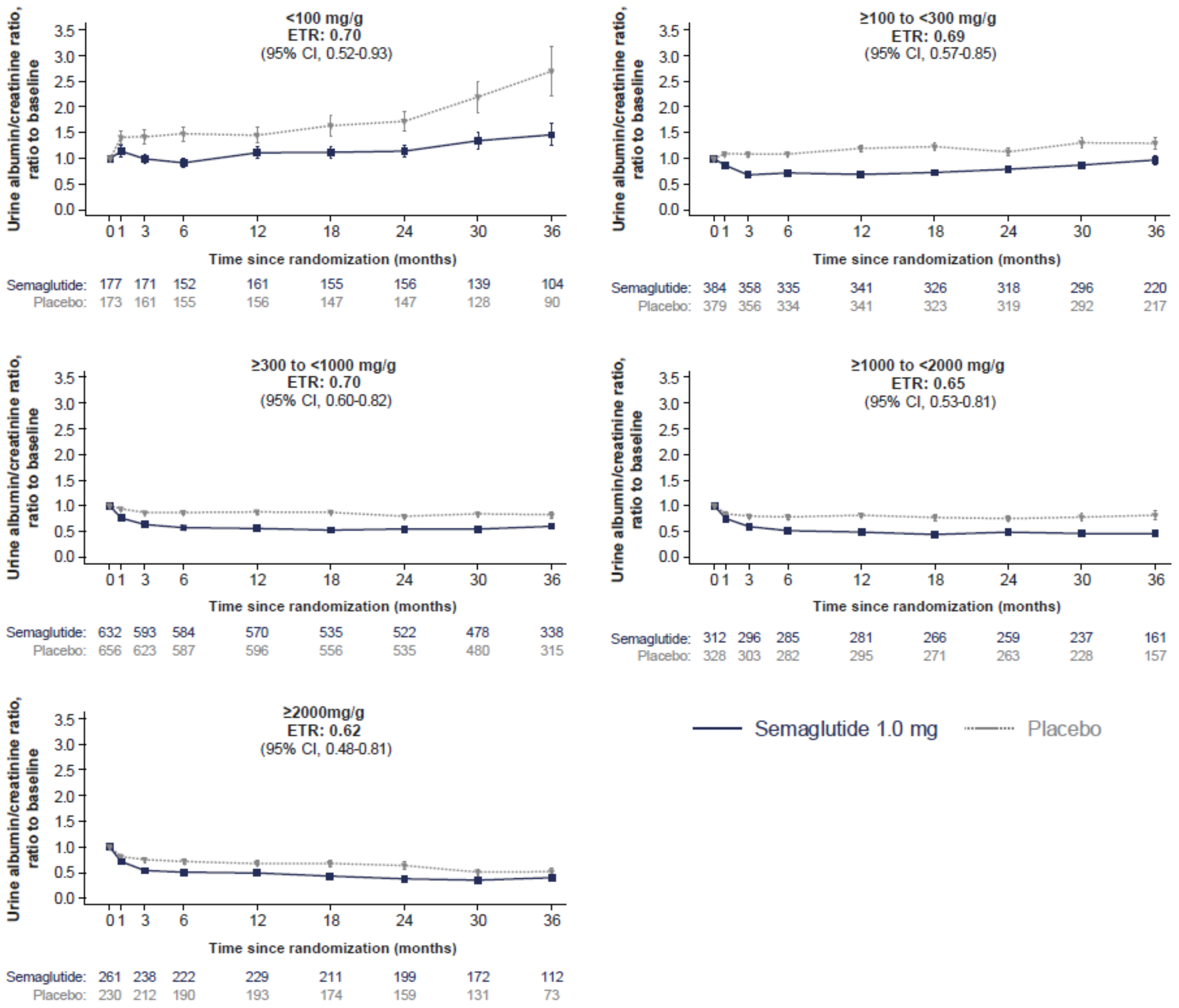

P for interaction was 0.94. Data are from the in-trial period. Error bars are +/− standard error of the mean on the logarithmic scale and back transformed to natural scale with the exponential. Numbers shown under the axes represent the number of participants contributing to the means. Responses at 24 months were analyzed using an ANCOVA with treatment, use of SGLT-2 inhibitor (yes/no) at baseline, baseline UACR subgroup and interaction between treatment and baseline UACR subgroup as fixed factors, and baseline value as covariate. ANCOVA, analysis of covariance; CI, confidence interval; ETR, estimated treatment ratio; SGLT2, sodium–glucose cotransporter-2; UACR, urine albumin-to-creatinine ratio.

FLOW trial investigators[1]

The number of patients enrolled by investigators is included in parentheses; site names and identification numbers are also provided.

Argentina: A. Porto, Glenny Corp. S.A, 951 (18); A. Oviedo, Medical Center of Diabetes and Nutrition, 952 (15); A. Elbert, Centro de Estudios Renales e Hipertensión Arterial, 953 (13); E. Gelersztein, Centro de Investigación Clínica, 954 (30); A. Chertkoff, Centro Diabetológico y Nuticional, 955 (14); A. Wassermann, FEPREVA, 956 (12); M.A. Quevedo, Centro Medico Dra. Laura Maffei e Investigacion Clínica Apli, 957 (26). **Australia:** R. MacIsaac, St Vincent's Hospital Fitzroy, 900 (6); S. Roger, Gosford Renal Research, 901 (21); R. Phoon, Westmead Hospital Northmead, 902 (7); P. Kerr, Monash Health Nephrology, 903 (6); A. Ajani, Monash University Clinical Trial Centre, 904 (1); S. J. Tan, The Royal Melbourne Hospital, 905 (5); D. Colquhoun, Core Research Centre, 906 (10); A. Mather, Royal North Shore Hospital, 907 (3). **Belgium:** P. Gillard, UZ Leuven - Endocrinology, 881 (10); C. De Block, UZ Antwerpen - UZA - Department of Endocrinology, 882 (6); C. Debroye, Universitair Ziekenhuis Brussel - Diabeteskliniek, 883 (6); F. Duyck, AZ Delta - Roeselare - Diabeteskliniek, 884 (5); J.M. Krzesinski, Centre Hospitalier Universitaire de Liège (CHU de Liège), 885 (5); B. Lapauw, UZ Gent - Thoracale-Vasculaire Heelkunde, 886 (6); V. Preumont, Hopital Saint Joseph-Saint Luc, 887 (5); C. Vercammen, Imeldaziekenhuis - Bonheiden - Department of Endocrinology, 888 (8). **Bulgaria:** Z. Kamenov, UMHAT Aleksandrovska, 835 (5); R. Bobeva, MHAT "Dr. Ivan Seliminski"-Sliven, 836 (11); G. Levterov, UMHAT "Kaspela", Department of Endocrinology and Metabolic Diseases, 837 (17); S. Gerilovska, Medical Center Synexus Sofia, 838 (8); N. Yabrudi, "Nader Yabrudi - ASMPVBE Individual practice", 839 (19); B. Stoyanovska-Elencheva, Medical Centre "Zdrave 1" OOD, 840 (8); Z. Nikitov, Medical Center "Nov Rehabilitatsionen Center" EOOD, 841 (7). **Brazil:** D. Franco, CPCLIN - Centro de Pesquisas Clínicas, 861 (16); G. Akerman Augusto, CPQuali Pesquisa Clínica Ltda, 862 (23); J. Salles, Instituto de Pesquisa Clinica (IPEC), 863 (10); L. Canani, Núcleo de Pesquisa Clínica do Rio Grande do Sul Ltda., 864 (27); M. Krakauer, Hospital e Maternidade Dr. Christovão da Gama S.A., 865 (10); M.J. Cerqueira, Instituto de Ensino e Pesquisa Clínica do Ceará Ltda, 866 (11); M. Riella, Instituto Pró-Renal Brasil, 867 (27). **Canada:** H. Bajaj, Dr. Harpreet Bajaj, 795 (11); R. Schlosser, LMC Clin Res Inc. Thornhill, 796 (18); B. Ajala, LMC Clin Res Inc. Calgary, 797 (10); N. Aggarwal, Aggarwal and Assoc Ltd., 798 (7); R. Dumas, Ctr de Rech Clin de Laval, 799 (17); D. O'Keefe, Commonwealth Medical Clinic, 800 (2); S. Peterson, Bluewater Clin Res Group,Inc, 801 (4); G. Tsoukas, Applied Med Inf Res, 802 (4); C. Tailor, LMC Research Inc. Ottawa, 803 (7); R. Tytus, Hamilton Med Res Group, 804 (11); R. Akhras, Centre Medical Acadie, 805 (8); H. Khandwala, LMC Endo Ctr (Etobicoke) Ltd, 806 (9); O. Steen, LMC Endo Centres Ltd. (Bayview), 808 (5); D. Carbonneau, Centre de Recherche Saint-Louis, 810 (3); J. Cha, Dr. James Cha, 811 (3); A. Steele, Lakeridge Health, 814 (2); G. Bailey, The Bailey Clinic, 815 (2); J. Berlingieri, JBN Medical Diagnostic Services Inc., 816 (1); P. Dzongowski, Milestone Research, 817 (4); D. Cherney, UHN-Toronto General Hospital, 822 (5); T. Elliott, BC Diabetes Canada, 823 (5); C. Kovacs, Eastern Health Authority, 824 (6); P. Hamet, Clinique de Recherche Medpharmgene Inc., 825 (7); B. Perkins, Mount Sinai Hospital, 826 (4); K. Tennankore, QE II Health - Pharmacy, 827 (1); A. Gupta, Dr Anil K Gupta Med Prof Corp, 830 (3). **China:** L. Ji, Peking University People's Hospital, 780 (3); Y. Li, Beijing Pinggu Hospital, 781 (5); G. Yuan, The Affiliated Hospital of Jiangsu University_Zhenjiang, 782 (11); X. Dong, Jinan Central Hospital, 783 (11). **France:** B. Cariou, Hôpital Nord Laënnec-Service d'Endocrinologie, 726 (10); S. Clavel, Groupe SOS Santé Hôtel Dieu, 727 (5); L. Marchand, Groupe Hospitalier Mutualiste des Portes du Sud, 728 (11); Y. Reznik, Chu de Caen - Svce Endoc, 729 (5); P. Moulin, Hôpital Cardiologique, 730 (4); E. Legrand, Centre Hospitalier d'Annonay, 731 (12); I. Benoit Tricaud, Centre Hospitalier Départemental La Roche sur Yon, 732 (9); P. Gourdy, Hôpital Rangueil, 733 (8); P. Zaoui, Hôpital Nord Michallon - Néphro, 734 (7); A. Monier, CH Louis Pasteur, 735 (5); I. Kazes, CHRU de Reims Hopital Maison Blanche, 736 (3). **Germany:** D. Dahl, Wendisch/Dahl Hamburg, 705 (12); M. Esser, Praxis Dr. med. M. Esser, 706 (6); T. Krüger, DaVita Clinical Research Deutschland GmbH, 707 (8); L. Rose, Institut für Diabetesforschung GmbH Münster - Dr. med. Rose, 708 (4); R. Schmieder, Medizinische Klinik 4, Universitätsklinikum

Erlangen, 709 (2); A. Segner, Segner, St. Ingbert, 711 (10); Y.H. Lee-Barkey, Dr. med. Young Hee Lee Barkey, 712 (9); S. Vidal, Diabetespraxis Mergentheim, 713 (11); T. Schürholz, InnoDiab Forschung GmbH, 714 (10). **Greece**: I. Boletis, General Hospital of Athens "Laiko" Nephrology Clinic, 680 (7); A. Raptis, University Hospital of Athens ATTIKON, 681 (4); L. Lanaras, General Hospital of Lamia, 682 (19); E. Pagkalos, "Thermi" Private Hospital, 683 (6); J. Doupis, Iatriko Athinon 'Palaiou Falirou', 684 (16); E. Bekiari, General Hospital of Thessaloniki "Ippokrateio" 685 (4); D. Goumenos, General University Hospital of Patras Nephrology Clinic, 686 (7); D. Papadopoulou, Genl Hosp of Thessaloniki, Papageorgiou Nephrology Clinic, 687 (2); K. Tziomalos, AHEPA General University Hospital, 688 (10); G. Karousos, Iatriko Psychicou Private Clinic, 689 (7); M. Somali, EUROMEDICA Gen Clinic The/ki, Endocrin, Metabolism, Diabetes, 691 (8); A. Markou, General Hospital of Athens 'G.Gennimatas' 692 (7); I. Zografou, "Ippokrateio" G.H. of Thessaloniki, 693 (4). **Hungary:** I. Wittmann, PTE-AOK II. Belgyogyaszati Klinika es Nephrologiai Centrum, 650 (10); P. Brasnyó, Siófoki Kórház, Diabetológiai Szakrendelés, 651 (8); J. Balla, Debreceni Egyetem Klinikai Központ Belgyógyászati Klinika, 652 (9); G. Petro, Debreceni Egyetem Klinikai Központ Belgyógyászati Klinika D épület, 653 (16); M. Baranyai, Markusovszky Egyetemi Oktatókórház, 655 (14); M. Dudás, Békés Megyei Központi Kórház, 656 (14); S. Vangel, Belinus Bt., 658 (27); L. Könyves, Lausmed Kft., 659 (10); S. Vasas, Borbánya Praxis E.Ü. Kft., 660 (17); T. Tanczer, MED-TIMA Kft., 661 (5). **India:** M. Shah, Madras Medical Mission Hospital, 600 (4); B.T. Anil, BGS Global Hospitals, 601 (3); A. Bhalla, Sir Ganga Ram Hospital, 602 (4); M.S. Gireesh, M S Ramaiah Memorial Hospital, 603 (2); N. Prasad, Sanjay Gandhi Postgraduate Institute of Medical Science, 604 (1); M. Sahay, Osmania General Hospital, 606 (14); T. Jamale, Seth GS Medical College and KEM Hospital, 607 (7); M. Jain, Apollo Multispeciality Hospital, Kolkata, 609 (1); M. Magdum, Poona Hospital and Research Centre, 610 (5); D. Dewan, Ajanta ResearchCentre, 612 (8); K. Dinesh, Max Super Speciality Hospital, Saket, 613 (8); H. Kumar, Amrita Institute of Medical Sciences & Research Centre, 614 (12); K. Jayakumar, Calicut Medical College, 615 (50); S. Murthy, Lifecare Hospital and Research Centre, 616 (38); K. Srikanth, Endolife Specialty Hospitals, 617 (40); S. Gupta, MV Hospital and Research Centre, 618 (34). **Israel:** G. Aharon-Hananel, Diabetes Unit Hadassah Ein Karem MC, 551 (10); J. Wainstein, Diabetes Clinic Wolfson MC, 552 (28); G. Chernin, Kaplan Medical Center Rehovot, 553 (11); I. Kenis, Meir Medical Center, 554 (12). **Italy**: R. Trevisan, Azienda Ospedaliera Papa Giovanni XXIII, 560 (20); E. Bosi and E. Setola, Istituto Scientifico San Raffaele, 561 (25); P. Fiorina, Azienda Ospedaliera Luigi Sacco, 562 (12); G. Penno, Presidio Ospedaliero Cisanello, 563 (12); G. Pugliese, Azienda Ospedaliero - Universitaria Sant'Andrea, 564 (10); C. Giordano, AOUPol.Giaccone UOC Malattie Endocrine, Ricambio e Nutrizione, 565 (4); E. Orsi, Ospedale Maggiore Policlinico UO Endocrinologia Diabetolgia, 566 (8); M.G. Cavallo, Azienda Ospealiero Universitaria Policlinico Umberto I, 567 (3). **Japan**: K. Tsuchida, Manda Memorial Hospital, 501 (19); T. Sasaki, Sasaki Hospital Internal Medicine, 502 (13); H. Seino, Seino Internal Medicine Clinic, 503 (16); T. Kawada, Kawada Clinic, 504 (9); T. Osonoi, Naka Kinen Clinic, 505 (14); H. Ohashi, Oyama East Clinic, 506 (15); Y. Shimizu, Shimizu Clinic Fusa, 507 (11); S. Mitomo, New Tokyo Heart Clinic, 508 (10); C. Nakamura, Yokohama Minoru Clinic, 509 (2); K. Takai, Shonan Takai Clinic, 510 (5); H. Maeda, H.E.C Science Clinic, 511 (13); H. Nishi, The University of Tokyo Hospital, 512 (2); Y. Nishida, Juntendo University Hospital Tokyo, 513 (1); M. Yamada, Tokyo Center Clinic, 514 (8); Y. Fukushima, Fukuwa Clinic, 515 (19); M. Kato, Kato Clinic of Internal Medicine, 516 (11); M. Sugawara, Sugawara Clinic, 517 (10); H. Araki, Fukui Prefectural Hospital Fukui, 518 (8); A. Nakagawa, Kanazawa Medical University Hospital, 519 (2); H. Onaka, Takatsuki Red Cross Hospital, 520 (16); A. Sueyoshi, Uji Tokushukai Medical Center, 521 (6); K. Kidokoro, Kawasaki Medical School Hospital, 522 (9); M. Tanabe, Fukuoka Tokushukai Hospital, 523 (11); H. Jinnouchi, Jinnouchi Hospital, 524 (13); S. Nakamura, Heiwadai Hospital, 525 (10); S. Hasumi, Nishiyamado Keiwa Hospital, 526 (3). **Mexico:** G. Gonzalez-Galvez, Inst. Jaliscience de Investigacion en Diabetes y Obesidad SC, 451 (19); C. Aguilar Salinas, Instituto Nacional de Nutricion - Unidad de Inv de Enf Metab, 452 (9); M. Morales de Teresa, Centro de Atención al Diabetico, 453 (19); R.M. Violante Ortiz, Centro de Estudios de Investigación Metabólicos y Cardio, 454 (17); J. Correa Rotter, Instituto Nacional de Ciencias Médicas y Nutrición Mexico City, 455 (8); S. Irizar Santana, Centro para el Desarrollo de la Medicina y la Asistencia, 456 (10). **Malaysia**: S.K. Lim, University Malaya Medical Centre, 481 (20); C.K. Yoon, Hospital Pulau

Pinang, 482 (15); F. Tan, Sarawak General Hospital, 483 (10); B.L. Goh, Hospital Serdang, 484 (11); C.L Loh, Hospital Raja Permaisuri Bainun Ipoh, 485 (12); N.S. Yusoff Azmi Merican, Hospital Sultanah Bahiyah, 486 (14). **Netherlands**: W.A. Bax, Noordwest Ziekenhuisgroep, 430 (5); D. van Raalte, Amsterdam UMC Lokatie VUMC, 431 (10); P.T. Luik, Meander Medisch Centrum, 432 (9); P.AM. de Vries, Ziekenhuis Groep Twente Almelo, 433 (8); J. Dorresteijn, Universitair Medisch Centrum Utrecht, 434 (4); M. AlHakim, EB Flevo Research BV, 435 (2); S. van Wissen, OLVG West, 436 (6); P. Kamphuisen, Tergooi, Locatie Hilversum, 437 (2); J. Verhave, Rijnstate Ziekenhuis, 438 (2). **Poland**: J. Gumprecht, Prywatny Gabinet Janusz Gumprecht, 915 (31); G. Majcher-Witczak, NZOZ WITAMED Poradnia Diabetologiczna, 917 (1); D. Mlodawska-Choluj, Centrum Medyczne "Diabetika" 919 (8); T. Stompor, Wojewodzki Szpital Specjalistyczny w Olsztynie, 920 (8); K. Ciechanowski, SPSK nr 2 PUM, Kl. Nefrologii, Transplantologii i Ch. Wewn., 921 (17); R. Malecki, Miedzyleski Szpital Specjalistyczny, Oddzial Nefrologiczny, 922 (6); K. Wasilewska, NZOZ Osteo-Medic S.C, 925 (7). **Russia**: M. Shamkhalova, National Medical Research Center of Endocrinology, 401 (8); L. Ruyatkina, Limited Law Company "Healthy Family" Medicine Center, 402 (16); G. Vagapova, PIH "Clin Hosp RZD-Medicina" former Kazan OJSC Rus Railways, 404 (7); M. Sergeeva-Kondrachenko, Penza Regional Clinical Hospital named after N.N. Burdenko, 405 (11); L. Kargina, Regional Clinical Cardiology Dispensary, 406 (15); A. Peskov, Ulianovsk Regional Clinical Hospital, 407 (12); E. Zhdanova, Vorornezh Regional Clinical Consultive-Diagnostic Centre, 408 (25); T. Lysenko, City Hospital #5, 409 (6); E. Frolova, Polyclinic #2 in Yoshkar-Ola, 410 (33); O. Mishchenko (Golinskaya, SAHI Kuzbass Hospital(former Regional Clinical Hospital), 413 (12); L. Belousova, Limited Liability Company "Energiya Zdoroviya", 414 (16); K. Astamirova, Aurora MedFort LLC, 415 (23); E. Antonova, Joint Stock Company "Modern Medical Technologies", 416 (29); Y. Pergaeva, Reg. State Budget Healthc. Inst. Regional Clinical Hospital, 418 (3); S. Palyutin, Solovyev Clinical Emergency Hospital, 419 (6); A. Sharkaeva, Medinet LLC, 420 (2); N. Krasnopeeva (Kabachkova), Road Clinical Hospital at Station Chelyabinsk, 421 (8); A. Tyugaeva, SHI Saratov City Clinical Hospital #9, 423 (7); E. Krasilnikova, Regional Endocrinological Dispensary, 425 (9); E. Romasheva, LLC «Endocrinolog», 426 (4); M. Sorokin, LLC RC Medical, 427 (18). **Slovakia**: E. Martinka, Narodny Endokrinologicky a Diabetologicky Ustav, 375 (16); A. Ilavska, MEDISPEKTRUM s.r.o., Diabetologicka ambulancia, 376 (11); L. Tomasova, Diabetologicka Ambulancia IN-DIA s.r.o., 377 (18); K. Porubska, DIABETIK s.r.o., 378 (7); D. Skripova, ARETEUS s.r.o., 379 (16); I. Buganova, Medivasa s.r.o., Diabetologicka Ambulancia, 380 (9); J. Rociakova, MUDr. Jana Rociakova s.r.o., Ambulancia vnutorneho lekarstva, 381 (10); J. Rosenberger, FMC - dialyzacne sluzby, s.r.o., 383 (5); A. Pavelcikova, FMC- Dialyzacne Sluzby, s.r.o., 384 (3); R. Smik, Diabetologicka Ambulancia MEDIKARD, s.r.o., 385 (5). **Spain**: J. Górriz, Hospital Clínico Universitario de Valencia, 340 (11); C. Castro, Hospital Universitario Doctor Peset, 341 (15); S. Cigarrán, Hospital Público da Mariña, 342 (14); C. Barrios, Hospital del Mar, 343 (7); M. Soler, Hospital Vall d'Hebron, 344 (14); J. Martins, Hospital Universitario de Getafe, 345 (11). **Thailand**: A. Sriwijitkamol, Siriraj Hospital Bangkoknoi, Bangkok, 310 (21); M. Phornphutkul, Maharaj Nakorn Chiang Mai Hospital, 311 (11); O. Supasyndh, Phramongkutklao Hospital-Nephrology, 312 (30); K. Kiattisunthorn, Siriraj Hospital-Nephro, 313 (13); P. Buranasupkajorn, King Chulalongkorn Memorial Hospital Bangkok, 314 (24); P. Susantitaphong, King Chulalongkorn Memorial Hospital-Division of Nephrology, 315 (10). **Turkey**: O. Helvaci, Gazi University Medical Faculty Nephrology, 285 (1); K. Ates, Ankara Universitesi Ibni Sina Hastanesi, 286 (4); I. Arikan, T.C. Saglik Bakanligi Pendik Egitim ve Arastirma Hastanesi, 287 (2); M. Arici, Hacettepe Universitesi Tip Fakultesi Nefroloji Bilim Dali, 288 (6); G. Suleymanlar, Akdeniz Universitesi Hastanesi Nefroloji Bilim Dali, 289 (7); H. Yazici, Istanbul Universitesi Istanbul Tip Fakultesi-Nefroloji, 290 (1); S. Alisir Ecder, Goztepe Egitim Arastirma Hastanesi Nefroloji, 291 (4); O. Gungor, Kahramanmaras Sutcu Imam University, 292 (13); E. Ok, Ege University Medical Faculty, 294 (9); N. Seyahi, Istanbul University Cerrahpasa Medical Faculty, 295 (6); N. Eren, Kocaeli University Nephrology Department, 296 (22); Y. Yenicerioglu, Aydin Adnan Menderes University Nephrology, 297 (9). **Ukraine**: O. Chabanna, CI "Zaporizhia City Clinical Hospital #10", 271 (28); S. Panina, SI "Institute of Medical and Social Problems of Disability", 273 (3); P. Semenovykh, SI "National Institute of Therapy n.a. L.T. Malaya of NAMSU", 274 (11); V. Leonidova, CNPI "Kharkiv City Clinical Hospital #27", 275 (10); O. Zinych, Institute of Endocrinology and Metabolism of

AMSU, 276 (30); O. Legun, Ivano-Frankivsk Regional Clinical Hospital, 277 (5); I. Kaydashev, CI "1st City Clinical Hospital of Poltava City Council", 279 (2). **United Kingdom:** S. Bellary, Diabetes Centre, Heartlands Hospital, Birmingham, 240 (9); J. Boyle, Glasgow Clinical Research Facility, 241 (4); M. Chen, St Georges Hospital, 242 (1); J. Mcknight, Western General Hospital, 243 (2); E. Pearson, Ninewells Hospital, 244 (6); S. Philip, Aberdeen Royal Infirmary, 245 (6); S. Sharma, The Diabetes Centre, 246 (10); A. Viljoen, Lister Hospital, 247 (6); J. Wakeling, Ely Bridge Surgery, 248 (5); H. Tindall, North Middlesex Hospital, 249 (8); A. Yousseif, Royal Free - Diabetes, 250 (5). **United States of America**: T. Aw, Valley Research, 102 (13); A. Guerrero, NE Clin Res of San Antonio, 104 (6); A. Arif, AA MRC LLC, 106 (8); J. Betts, Research Management, Inc, 108 (1); R. Busch, Albany Medical College – Endo, 109 (24); C. Desouza, Univ of Nebraska Medical CTR, 110 (3); L. Gonzalez-Orozco, Clinical Invest Special Gurnee, 112 (4); C. Dukes, Sun Research Institute, 113 (9); J. Hammoud, Elite Research Center, 114 (25); P. Houser, Amherst Family Practice P.C., 115 (4); T. Jackson, Heritage Valley Medical Group Inc, 116 (7); Z. Kayali, Inland Empire Clin Trials LLC, 117 (4); L. Young, UNC Diabetes Care Center Chapel Hill, 118 (4); E. Klein, Capital Clin Res Ctr,LLC, 119 (6); I. Lingvay, UT Southwestern Med Cntr, 120 (4); P. Nicol, The Diabetes Center, LLC, 121 (1); S. Smith, Burke Primary Care, 122 (1); B. Snyder, Southgate Medical Group, LLP, 123 (6); E. Soroka, Saviers Medical Group, 124 (4); C. Wysham, MultiCare Inst for Res & Innov, 125 (6); A. Akyea-Djamson, Metropolitan Cardiovascular Consultants, LLC, 126 (3); C. Arauz-Pacheco, Carlos Arauz-Pacheco MD, PA, 127 (8); A. Horeish, California Inst of Renal Res, 129 (5); A. Chang, John Muir Physician Network, 130 (5); L. Connery, Intend Research, 131 (5); W. Gandy Jr, Ellipsis Group, 132 (4); M. Gilbert, University of Vermont Medical Center, 133 (3); G. Hernandez, MedResearch Inc, 134 (4); M. Hewitt, Northern Pines Hlth Ctr, PC, 135 (2); D. Huffman, Univ Diab & Endo Consultants, 136 (3); C. Lovell, York Clinical Research LLC, 137 (10); N. Masani, NYU Langone Neph Associates, 138 (10); J. McGill, Washington University St. Louis, 139 (1); I. Siddiqui, Simcare Medical Research, LLC, 140 (6); R. Perez, Encore Medical Research LLC, 141 (16); J. Reed III, Endocrine Research Solutions, 142 (3); K. Sivalingam, First Valley Med Grp Lancaster, 143 (6); J. London, San Fernando Valley Hlth Inst, 144 (1); S. Saha, Cincinnati Veterans Affairs Medical Center, 145 (2); A. Awad, Clinical Research Consultants, LLC, 147 (7); L. Billings, NorthShore Univ Hlth Sys, 148 (14); K. Blaze, South Broward Research LLC, 149 (3); D. Stricklin, Four Rivers Clinical Research Inc, 151 (5); C. Brinson, Central Texas Clinical Research, 152 (2); J. Bornfreund, Family Medicine of SayeBrook LLC, 153 (3); N. McCall, Vanderbilt Medical Center Nashville, 154 (4); R. Buynak, Velocity Clin. Res Valparaiso, 155 (10); K. Cannon, Accellacare, 156 (6); N. Daboul, Advanced Med Res Maumee, 158 (9); I. Acosta, San Marcus Res Clin Miami Lakes, 159 (16); J. Diaz, Palmetto Medical General Plaza, 160 (4); G. Greenwood, Brookview Hills Research Associates, LLC, 162 (19); T. Hart, Dr. Terence Hart, 163 (1); S. Hasan, Suncoast Clinical Research, Inc., 164 (5); A. Iranmanesh, VA Medical Center Salem, 165 (8); R. Koilpillai, Clinical Research of Central Florida_Winter Haven, 167 (12); K. Latif, AM Diabetes and Endocrinology Center, 168 (5); M. Lawrence, Carteret Medical Group, 169 (9); C. Chen, VA Long Beach Healthcare System, 170 (1); I. Marar, West Broadway Clinic, 172 (4); C. East, Baylor Jack and Jane Hamilton, 173 (2); R. Patel, Lycoming Internal Medicine, Inc., 175 (2); S. Rovner, Academy of Diabetes, Thyroid & Endocrine, P.A., 177 (5); G. Seco, Homestead Associates in Research, 178 (5); Z. Shaikh, Southwest Clinical Trials, 179 (4); H. Traylor, Whiteville Medical Associates, 180 (1); F. Zieve, McGuire VA Medical Center, 181 (1); V. Aroda, Brigham & Women's Hospital, 182 (3); A. Ahmed, Apex Medical Research Inc, 183 (9); M. Biscoveanu, Northeast Endocrine Metabolic Associates, 186 (3); M. Budoff, Lundquist Inst-Biomed Innovtn, 187 (4); M. El-Shahawy, Academic Medical Research Institute, 188 (7); J. Garza, VIP Trials, 190 (4); L. Hanson, Hanson Clinical Research Center, 191 (6); J. Reddy, Sierra Clinical Research, 194 (1); H. Rodriguez, Life Spring Research, 195 (11); A. Shrestha, Northeast Research Institute, 196 (10); M. Bernardo, Southwest Houston Research Ltd, 197 (10); T. Christensen, Main Street Physician's Care, 198 (2); P. Denker, BayCare Medical Group - Endocrinology, 200 (10); S. Dua, Valley Renal Medical Group Research, 201 (5); M. El Shahawy, Cardiovascular Ctr of Sarasota, 202 (5); A. Farias, International Physicians Research, 203 (3); R. Fernando, VA Loma Linda Hlthcr Sys, 204 (20); A. Jamal, N America Res Inst - San Dimas, 205 (17); M. Jardula, Desert Oasis Hlthcr Med Group, 206 (10); M. Moustafa, SC Nephrology and Hypertension, 209 (1); J. Posada, Horizon Rs Grp, Coral Gbls, 210 (4); W. Sanchez, Floridian

Clinical Research, LLC, 211 (9); D. Vega, Juno Research, LLC Houston, 212 (6); J. Kumar, Renal Medicine Associates, 214 (9); S. Sultan, Northeast Research Institute, 216 (22); S. Bansal, Univ of Texas Hlth Science Cntr, 217 (5); S. Benjamin, Universal Research Group, 218 (3); O. Brusco, Osvaldo A. Brusco MD PA, 219 (13); J. Wayne, Clinical Trials Research Sacramento, 220 (5); R. Gaona, Briggs Clinical Research, LLC, 221 (7); O. Degani, North Suburban Nephrology LLC, 222 (4); C. Figueroa, Cesar Figueroa M.D. P.A., 223 (6); M. Montero, Eastern Nephr Assoc, PLLC, 224 (6); N. Rasouli, Rocky Mount Reg VA Med-DN, 226 (5); L. Zemel, Creekside Endocrine Associates, PC, 228 (9); G. Fadda, California Institute of Renal Research, 229 (3); Y. Shlesinger, NorCal Endocrinology and Internal Medicine, 230 (10); D. Charytan, NYU Nephrology Associates, 231 (1); D. Butuk, Solaris Clinical Research, 233 (4); S. Stringam, Saltzer Medical Group Research, 236 (5); V. Espinosa, Texas Diabetes & Endocrinology, P.A. Austin, 237 (3); D. Brandon, California Research Foundation, 238 (9); H. Shea, Research Institute of Dallas, 239 (1); D. Cheung, Long Beach Center for Clinical Research, 960 (1); Y. Sanchez, Oceane 7 Medical & Research Center, Inc., 962 (7); Y. Kudva, Mayo Clinic, 963 (3); S. Beddhu, University of Utah School of Medicine, 964 (1). **South Africa**: B. Rayner, Prof Rayner, 355 (17); Q. Bhorat, Soweto Clinical Trial Centre, 356 (1); A. Badat, Wits Bara Clinical Trial Site, 357 (30); H. Makan, Hemant Makan, 358 (13); M. Joshi, Diabetes Care Centre & CDE Centre, 359 (10); M. Basson, Karl Bremer Hospital, 362 (7); V. Naidoo, Precise Clinical Solutions (Pty) Ltd, 363 (7); A. Amod, Dr A Amod, 364 (16).

References

1. Perkovic V, Tuttle KR, Rossing P, et al. Effects of semaglutide on chronic kidney disease in patients with type 2 diabetes. *N Engl J Med*. 2024;391(2):109–121. doi: 10.1056/NEJMoa2403347.
2. Rossing P, Baeres FMM, Bakris G, et al. The rationale, design and baseline data of FLOW, a kidney outcomes trial with once-weekly semaglutide in people with type 2 diabetes and chronic kidney disease. *Nephrol Dial Transplant*. 2023;38(9):2041–2051. doi: 10.1093/ndt/gfad009.
