# Supplementary material for: Kidney and Survival Outcomes with Semaglutide by CKD Severity in the FLOW Trial
Source: Clin J Am Soc Nephrol. 2026 Feb 18;21(5):841–51. doi: 10.2215/CJN.0000000974 (PMC13143484; doi:10.2215/CJN.0000000974)
Supplement: Supplementary file 2 [file cjasn-21-841-s002.pdf]

ASN American Society of Nephrology | JASN Journal of the American Society of Nephrology | KIDNEY360 Accessing Our World From Every Angle | CJASN Clinical Journal of the American Society of Nephrology

ASN Journal Disclosure Form

As per ASN journal policy, I have disclosed any financial relationships or commitments I have held in the past 36 months as included below. I have listed my Current Employer below to indicate there is a relationship requiring disclosure. If no relationship exists, my Current Employer is not listed.

M. Arici reports the following:
Employer: Hacettepe University Medical Faculty; and Honoraria: Amgen, Astellas, Astra Zeneca, Bayer, Boehringer Ingelheim, Menarini, Novo Nordisk, Recordati, Sandoz, Sanofi.

I understand that the information above will be published within the journal article, if accepted, and that failure to comply and/or to accurately and completely report the potential financial conflicts of interest could lead to the following: 1) Prior to publication, article rejection, or 2) Post-publication, sanctions ranging from, but not limited to, issuing a correction, reporting the inaccurate information to the authors' institution, banning authors from submitting work to ASN journals for varying lengths of time, and/or retraction of the published work.

Name: Mustafa Arici
Manuscript ID: CJASN-2025-001670R1
Manuscript Title: Kidney and Survival Outcomes with Semaglutide by Chronic Kidney Disease Severity in the FLOW Trial
Date of Completion: November 22, 2025
Disclosure Updated Date: May 24, 2025

ASN American Society of Nephrology | JASN Journal of the American Society of Nephrology | KIDNEY360 Accessing Our World From Every Angle | CJASN Clinical Journal of the American Society of Nephrology

ASN Journal Disclosure Form

As per ASN journal policy, I have disclosed any financial relationships or commitments I have held in the past 36 months as included below. I have listed my Current Employer below to indicate there is a relationship requiring disclosure. If no relationship exists, my Current Employer is not listed.

H. Bosch-Traberg reports the following:
Employer: Novo Nordisk A/S; and Ownership Interest: Novo Nordisk A/S.

I understand that the information above will be published within the journal article, if accepted, and that failure to comply and/or to accurately and completely report the potential financial conflicts of interest could lead to the following: 1) Prior to publication, article rejection, or 2) Post-publication, sanctions ranging from, but not limited to, issuing a correction, reporting the inaccurate information to the authors' institution, banning authors from submitting work to ASN journals for varying lengths of time, and/or retraction of the published work.

Name: Heidrun Bosch-Traberg
Manuscript ID: CJASN-2025-001670R1
Manuscript Title: Kidney and Survival Outcomes with Semaglutide by Chronic Kidney Disease Severity in the FLOW Trial
Date of Completion: November 24, 2025
Disclosure Updated Date: November 24, 2025

ASN American Society of Nephrology | JASN Journal of the American Society of Nephrology | KIDNEY360 Accessing Our World From Every Angle | CJASN Clinical Journal of the American Society of Nephrology

ASN Journal Disclosure Form

As per ASN journal policy, I have disclosed any financial relationships or commitments I have held in the past 36 months as included below. I have listed my Current Employer below to indicate there is a relationship requiring disclosure. If no relationship exists, my Current Employer is not listed.

D. Cherney reports the following:
Employer: Toronto General Hospital; Consultancy: Boehringer Ingelheim-Lilly, Merck, AstraZeneca, Sanofi, Mitsubishi-Tanabe, Abbvie, Janssen, AMGEN, Bayer, Prometic, BMS, Maze, Gilead, CSL-Behring, Otsuka, Novartis, Youngene, Lexicon, Inversago, GSK, Biobridge, Vantage, Altimmune and Novo-Nordisk; Research Funding: Boehringer Ingelheim-Lilly, Merck, Janssen, Sanofi, AstraZeneca, CSL-Behring, Lexicon, Novo-Nordisk, Bayer.; Honoraria: Boehringer Ingelheim-Lilly, Merck, AstraZeneca, Sanofi, Mitsubishi-Tanabe, Abbvie, Janssen, AMGEN, Bayer, Prometic, BMS, Maze, Gilead, CSL-Behring, Otsuka, Novartis, Youngene, Lexicon, Inversago, GSK, Biobridge, Vantage, Altimmune and Novo-Nordisk; and Advisory or Leadership Role: Boehringer Ingelheim-Lilly, Merck, AstraZeneca, Lexicon, Janssen, Bayer, BMS, Maze, CSL-Behring, Novartis, Novo-Nordisk, AMGEN, Roche.

I understand that the information above will be published within the journal article, if accepted, and that failure to comply and/or to accurately and completely report the potential financial conflicts of interest could lead to the following: 1) Prior to publication, article rejection, or 2) Post-publication, sanctions ranging from, but not limited to, issuing a correction, reporting the inaccurate information to the authors' institution, banning authors from submitting work to ASN journals for varying lengths of time, and/or retraction of the published work.

Name: David Cherney
Manuscript ID: CJASN-2025-001670R1
Manuscript Title: Kidney and Survival Outcomes with Semaglutide by Chronic Kidney Disease Severity in the FLOW Trial
Date of Completion: January 7, 2026
Disclosure Updated Date: January 7, 2026

ASN American Society of Nephrology | JASN Journal of the American Society of Nephrology | KIDNEY360 Accessing Our World From Every Angle | CJASN Clinical Journal of the American Society of Nephrology

ASN Journal Disclosure Form

As per ASN journal policy, I have disclosed any financial relationships or commitments I have held in the past 36 months as included below. I have listed my Current Employer below to indicate there is a relationship requiring disclosure. If no relationship exists, my Current Employer is not listed.

R. Correa-Rotter reports the following:
Employer: Instituto Nacional de Ciencias Médicas y Nutrición Salvador Zubiran, MEXICO and; Universidad Nacional Autónoma de México, MEXICO; Consultancy: Astra Zeneca, GSK, Boheringer Ingelheim, Bayer, Chinook, Novonordisk; Research Funding: Astra Zeneca, Novonordisk, Roche, Chinook, Lilly; Honoraria: Amgen, Astra Zeneca, Boehringer Ingelheim, Sanofi, Bayer, Amgen, Lilly; Advisory or Leadership Role: National Leader ASCEND study, GSK; National Leader and International Expert Forum FLOW study, Novonordisk, membership steering Committee FINE-REAL, Bayer. Editorial Board Nefrologia Latinoamericana, American Journal of Kidney Diseases, and Associate Editor: Blood Purification. Member of the Steering Committee of the World Kidney Day organization.; Speakers Bureau: Amgen, Astra Zeneca, Boehringer Ingelheim, Abbvie, Sanofi, Bayer, Novonordisk, Lilly; and Other Interests or Relationships: Member of ASN, of International Society of Nephrology; Member of National Kidney Foundation; Member Mexican Institute for Research in Nephrology; Member Latin American Society of Nephrology and Hypertension; Member ERA.

I understand that the information above will be published within the journal article, if accepted, and that failure to comply and/or to accurately and completely report the potential financial conflicts of interest could lead to the following: 1) Prior to publication, article rejection, or 2) Post-publication, sanctions ranging from, but not limited to, issuing a correction, reporting the inaccurate information to the authors' institution, banning authors from submitting work to ASN journals for varying lengths of time, and/or retraction of the published work.

Name: Ricardo Correa-Rotter
Manuscript ID: CJASN-2025-001670R1
Manuscript Title: Kidney and Survival Outcomes with Semaglutide by Chronic Kidney Disease Severity in the FLOW Trial
Date of Completion: November 21, 2025
Disclosure Updated Date: November 21, 2025

ASN American Society of Nephrology | JASN Journal of the American Society of Nephrology | KIDNEY360 Accessing Our World From Every Angle | CJASN Clinical Journal of the American Society of Nephrology

ASN Journal Disclosure Form

As per ASN journal policy, I have disclosed any financial relationships or commitments I have held in the past 36 months as included below. I have listed my Current Employer below to indicate there is a relationship requiring disclosure. If no relationship exists, my Current Employer is not listed.

J. Gumprecht reports the following:
Employer: Medical University of Silesia Poland; Consultancy: Novo Nordisk, Eli Lilly, Boehringer Ingelheim, Sanofi, Berlin Chemie, Astra Zeneca, Bayer, Polpharma, Servier, Roche, Abbott, Bioton, Merck, Dexcom, Medtronic, Adamed, Alfasigma; Research Funding: Novo Nordisk, Eli Lilly, Bayer; Honoraria: Novo Nordisk, Eli Lilly, Boehringer Ingelheim, Sanofi, Berlin Chemie, Astra Zeneca, Bayer, Polpharma, Servier, Roche, Abbott, Bioton, Merck, Dexcom, Medtronic, Adamed, Alfasigma; and Speakers Bureau: Novo Nordisk, Eli Lilly, Boehringer Ingelheim, Sanofi, Berlin Chemie, Astra Zeneca, Bayer, Polpharma, Servier, Roche, Abbott, Bioton, Merck, Dexcom, Medtronic, Adamed, Alfasigma.

I understand that the information above will be published within the journal article, if accepted, and that failure to comply and/or to accurately and completely report the potential financial conflicts of interest could lead to the following: 1) Prior to publication, article rejection, or 2) Post-publication, sanctions ranging from, but not limited to, issuing a correction, reporting the inaccurate information to the authors' institution, banning authors from submitting work to ASN journals for varying lengths of time, and/or retraction of the published work.

Name: Janusz Gumprecht
Manuscript ID: CJASN-2025-001670R1
Manuscript Title: Kidney and Survival Outcomes with Semaglutide by Chronic Kidney Disease Severity in the FLOW Trial,
Date of Completion: December 2, 2025
Disclosure Updated Date: December 2, 2025

ASN
American Society of Nephrology
JASN
Journal of the American Society of Nephrology
KIDNEY360
Accessing Our World From Every Angle
CJASN
Clinical Journal of the American Society of Nephrology

ASN Journal Disclosure Form

As per ASN journal policy, I have disclosed any financial relationships or commitments I have held in the past 36 months as included below. I have listed my Current Employer below to indicate there is a relationship requiring disclosure. If no relationship exists, my Current Employer is not listed.

O. Jeppesen reports the following:
Employer: Novo Nordisk A/S; and Ownership Interest: Novo Nordisk A/S.

I understand that the information above will be published within the journal article, if accepted, and that failure to comply and/or to accurately and completely report the potential financial conflicts of interest could lead to the following: 1) Prior to publication, article rejection, or 2) Post-publication, sanctions ranging from, but not limited to, issuing a correction, reporting the inaccurate information to the authors' institution, banning authors from submitting work to ASN journals for varying lengths of time, and/or retraction of the published work.

Name: Ole K. Jeppesen
Manuscript ID: CJASN-2025-001670R1
Manuscript Title: Kidney and Survival Outcomes with Semaglutide by Chronic Kidney Disease Severity in the FLOW Trial
Date of Completion: December 2, 2025
Disclosure Updated Date: December 2, 2025

ASN American Society of Nephrology | JASN Journal of the American Society of Nephrology | KIDNEY360 Accessing Our World From Every Angle | CJASN Clinical Journal of the American Society of Nephrology

ASN Journal Disclosure Form

As per ASN journal policy, I have disclosed any financial relationships or commitments I have held in the past 36 months as included below. I have listed my Current Employer below to indicate there is a relationship requiring disclosure. If no relationship exists, my Current Employer is not listed.

N. Kashihara reports the following:
Employer: Kawasaki Medical School; Consultancy: Astrazeneca, Novartis, Zeria; Research Funding: AstraZeneka, Boringer ,Daiichi-Sankyo,Otsuka,Novartis,Bayer; Honoraria: Boringer, Otsuka,Astrazeneca,Novartis,Bayer; Advisory or Leadership Role: Astrazeneca,Novartis, Boringer, Kyowa Kirin; Speakers Bureau: Astrazeneca,Novartis, Boringer, Kyowa Kirin; and Other Interests or Relationships: Japan Kidney Association; Japanese Society of Nephrology.

I understand that the information above will be published within the journal article, if accepted, and that failure to comply and/or to accurately and completely report the potential financial conflicts of interest could lead to the following: 1) Prior to publication, article rejection, or 2) Post-publication, sanctions ranging from, but not limited to, issuing a correction, reporting the inaccurate information to the authors' institution, banning authors from submitting work to ASN journals for varying lengths of time, and/or retraction of the published work.

Name: Naoki Kashihara
Manuscript ID: CJASN-2025-001670R1
Manuscript Title: Kidney and Survival Outcomes with Semaglutide by Chronic Kidney Disease Severity in the FLOW Trial,
Date of Completion: January 7, 2026
Disclosure Updated Date: January 7, 2026

ASN American Society of Nephrology
JASN Journal of the American Society of Nephrology
KIDNEY360 Accessing Our World From Every Angle
CJASN Clinical Journal of the American Society of Nephrology

ASN Journal Disclosure Form

As per ASN journal policy, I have disclosed any financial relationships or commitments I have held in the past 36 months as included below. I have listed my Current Employer below to indicate there is a relationship requiring disclosure. If no relationship exists, my Current Employer is not listed.

K. Mahaffey reports the following:
Employer: Stanford University School of Medicine; Consultancy: Applied Therapuetics, Bayer, BMS, BridgeBio, Eli Lilly, Elsevier, Fosun Pharma, Human, J&J, Moderna, Myokardia, Novartis, Novo Nordisk, Phasebio, Portola, Quidel, Scleroderma Foundation, Theravance; Ownership Interest: Human, Medeloop, Precordior, Regencor; and Research Funding: ACC, AHA, Apple Inc, Bayer, CIRM, CSL Behring, Cytokinetics, Eidos, Element, Ferring, Gilead, Google (Verily), Idorsia, J&J, Luitpold, Myovant, Novartis, PAC-12, Precordior, Sanifit, St. Jude Medical.

I understand that the information above will be published within the journal article, if accepted, and that failure to comply and/or to accurately and completely report the potential financial conflicts of interest could lead to the following: 1) Prior to publication, article rejection, or 2) Post-publication, sanctions ranging from, but not limited to, issuing a correction, reporting the inaccurate information to the authors' institution, banning authors from submitting work to ASN journals for varying lengths of time, and/or retraction of the published work.

Name: Kenneth W. Mahaffey
Manuscript ID: CJASN-2025-001670R1
Manuscript Title: Kidney and Survival Outcomes with Semaglutide by Chronic Kidney Disease Severity in the FLOW Trial
Date of Completion: December 1, 2025
Disclosure Updated Date: December 1, 2025

ASN American Society of Nephrology | JASN Journal of the American Society of Nephrology | KIDNEY360 Accessing Our World From Every Angle | CJASN Clinical Journal of the American Society of Nephrology

ASN Journal Disclosure Form

As per ASN journal policy, I have disclosed any financial relationships or commitments I have held in the past 36 months as included below. I have listed my Current Employer below to indicate there is a relationship requiring disclosure. If no relationship exists, my Current Employer is not listed.

J. Mann reports the following:
Employer: KfH Nierenzentrum; Consultancy: Bayer, Novo Nordisk; Research Funding: Novo Nordisk, Sanofi, Bayer, ICON, Parexel, Cytel, Iqvia, Fortrea; Honoraria: Bayer, Novo Nordisk, Sanofi, Hexal, ICON, Parexel, Cytel, Iqvia, WCG; and Speakers Bureau: Bayer, Hexal, Novo Nordisk.

I understand that the information above will be published within the journal article, if accepted, and that failure to comply and/or to accurately and completely report the potential financial conflicts of interest could lead to the following: 1) Prior to publication, article rejection, or 2) Post-publication, sanctions ranging from, but not limited to, issuing a correction, reporting the inaccurate information to the authors' institution, banning authors from submitting work to ASN journals for varying lengths of time, and/or retraction of the published work.

Name: Johannes F. Mann
Manuscript ID: CJASN-2025-001670R1
Manuscript Title: Kidney and Survival Outcomes with Semaglutide by Chronic Kidney Disease Severity in the FLOW Trial
Date of Completion: November 22, 2025
Disclosure Updated Date: July 23, 2025

ASN American Society of Nephrology | JASN Journal of the American Society of Nephrology | KIDNEY360 Accessing Our World From Every Angle | CJASN Clinical Journal of the American Society of Nephrology

ASN Journal Disclosure Form

As per ASN journal policy, I have disclosed any financial relationships or commitments I have held in the past 36 months as included below. I have listed my Current Employer below to indicate there is a relationship requiring disclosure. If no relationship exists, my Current Employer is not listed.

M. Mayrdorfer reports the following:
Employer: Novo Nordisk; and Ownership Interest: Novo Nordisk.

I understand that the information above will be published within the journal article, if accepted, and that failure to comply and/or to accurately and completely report the potential financial conflicts of interest could lead to the following: 1) Prior to publication, article rejection, or 2) Post-publication, sanctions ranging from, but not limited to, issuing a correction, reporting the inaccurate information to the authors' institution, banning authors from submitting work to ASN journals for varying lengths of time, and/or retraction of the published work.

Name: Manuel Mayrdorfer
Manuscript ID: CJASN-2025-001670R1
Manuscript Title: Kidney and Survival Outcomes with Semaglutide by Chronic Kidney Disease Severity in the FLOW Trial
Date of Completion: January 7, 2026
Disclosure Updated Date: January 7, 2026

ASN American Society of Nephrology | JASN Journal of the American Society of Nephrology | KIDNEY360 Accessing Our World From Every Angle | CJASN Clinical Journal of the American Society of Nephrology

ASN Journal Disclosure Form

As per ASN journal policy, I have disclosed any financial relationships or commitments I have held in the past 36 months as included below. I have listed my Current Employer below to indicate there is a relationship requiring disclosure. If no relationship exists, my Current Employer is not listed.

V. Perkovic reports the following:
Employer: University of New South Wales; Consultancy: AstraZeneca, Bayer, Biogen, Boehringer Ingelheim, Chinook, GlaxoSmithKline, Guard therapeutics, Incyte, Janssen, Mineralys, Novo Nordisk, Novartis, Otsuka, Shanxi Micot, Travere, Tricida, Vifor;; Ownership Interest: George Clinical; Honoraria: Steering committee, data monitoring committee or advisory board roles or for scientific presentations from AstraZeneca, Bayer, Biogen, Boehringer Ingelheim, Chinook, GlaxoSmithKline, Guard therapeutics, Incyte, Janssen, Mineralys, Novo Nordisk, Novartis, Otsuka, Shanxi Micot, Travere, Tricida, Vifor;; and Advisory or Leadership Role: Board member of Kidney Health Australia and St Vincents Health Australia.

I understand that the information above will be published within the journal article, if accepted, and that failure to comply and/or to accurately and completely report the potential financial conflicts of interest could lead to the following: 1) Prior to publication, article rejection, or 2) Post-publication, sanctions ranging from, but not limited to, issuing a correction, reporting the inaccurate information to the authors' institution, banning authors from submitting work to ASN journals for varying lengths of time, and/or retraction of the published work.

Name: Vlado Perkovic
Manuscript ID: CJASN-2025-001670R1
Manuscript Title: Kidney and Survival Outcomes with Semaglutide by Chronic Kidney Disease Severity in the FLOW Trial
Date of Completion: November 23, 2025
Disclosure Updated Date: July 6, 2025

ASN American Society of Nephrology
JASN Journal of the American Society of Nephrology
KIDNEY360 Accessing Our World From Every Angle
CJASN Clinical Journal of the American Society of Nephrology

ASN Journal Disclosure Form

As per ASN journal policy, I have disclosed any financial relationships or commitments I have held in the past 36 months as included below. I have listed my Current Employer below to indicate there is a relationship requiring disclosure. If no relationship exists, my Current Employer is not listed.

R. Pratley reports the following:
Employer: AdventHealth Translational Research Institute; Consultancy: Abbott Labs; AbbVie; Altanine; Amgen; AstraZeneca Pharma LP; Bayer AG; Bayer HealthCare Pharma; BI Pharma; Corcept; Eli Lilly & Co; Endogenex; F Hoffman-La Roche; Gasherbrum Bio; GetzPharma; Hanmi Pharma Co; Lexicon Pharma; Lilly USA; Novo; Pfizer; Recordati Rare Diseases; Regeneron Pharma; Response Pharma; Rona Therapeutics; Scholar Rock; Sun Pharma Industries; Third Rock Ventures; Verdiva Bio Dev. Thru 12/31/23 pd to Dr. Pratley's employer AdventHealth, after 1/1/24 to Dr. Pratley; Ownership Interest: Altanine, Inc.; Research Funding: Research grants from Abbott Laboratories; AstraZeneca AB; AstraZeneca Pharmaceuticals LP; Biomea Fusion; Boehringer Ingelheim International GmbH; Carmot Therapeutics; Dompe; Endogenex, Inc.; Eli Lilly & Co; Fractyl; Lexicon Pharmaceuticals; Metavention; National Institutes of Health; Novo Nordisk; and Sanofi. All payments are made directly to Dr. Pratley's employer (AdventHealth, a nonprofit corporation).; and Speakers Bureau: Lilly USA LLC; Novo Nordisk.

I understand that the information above will be published within the journal article, if accepted, and that failure to comply and/or to accurately and completely report the potential financial conflicts of interest could lead to the following: 1) Prior to publication, article rejection, or 2) Post-publication, sanctions ranging from, but not limited to, issuing a correction, reporting the inaccurate information to the authors' institution, banning authors from submitting work to ASN journals for varying lengths of time, and/or retraction of the published work.

Name: Richard E. Pratley
Manuscript ID: CJASN-2025-001670R1
Manuscript Title: "Kidney and Survival Outcomes with Semaglutide by Chronic Kidney Disease Severity in the FLOW Trial"
Date of Completion: November 25, 2025
Disclosure Updated Date: November 25, 2025

ASN American Society of Nephrology
JASN Journal of the American Society of Nephrology
KIDNEY360 Accessing Our World From Every Angle
CJASN Clinical Journal of the American Society of Nephrology

ASN Journal Disclosure Form

As per ASN journal policy, I have disclosed any financial relationships or commitments I have held in the past 36 months as included below. I have listed my Current Employer below to indicate there is a relationship requiring disclosure. If no relationship exists, my Current Employer is not listed.

G. Pugliese has nothing to disclose.

I understand that the information above will be published within the journal article, if accepted, and that failure to comply and/or to accurately and completely report the potential financial conflicts of interest could lead to the following: 1) Prior to publication, article rejection, or 2) Post-publication, sanctions ranging from, but not limited to, issuing a correction, reporting the inaccurate information to the authors' institution, banning authors from submitting work to ASN journals for varying lengths of time, and/or retraction of the published work.

Name: Giuseppe Pugliese
Manuscript ID: CJASN-2025-001670R1
Manuscript Title: Kidney and Survival Outcomes with Semaglutide by Chronic Kidney Disease Severity in the FLOW Trial
Date of Completion: January 8, 2026
Disclosure Updated Date: January 8, 2026

ASN American Society of Nephrology | JASN Journal of the American Society of Nephrology | KIDNEY360 Accessing Our World From Every Angle | CJASN Clinical Journal of the American Society of Nephrology

ASN Journal Disclosure Form

As per ASN journal policy, I have disclosed any financial relationships or commitments I have held in the past 36 months as included below. I have listed my Current Employer below to indicate there is a relationship requiring disclosure. If no relationship exists, my Current Employer is not listed.

B. Rayner reports the following:
Employer: Division of Nephrology and Hypertension, University of Cape Town; Research Funding: NovoNordisk, Astra-zeneca, Roche; and Honoraria: Servier; Boehringer-Ingelheim; Novartis; Astra-Zeneca; Bayer.

I understand that the information above will be published within the journal article, if accepted, and that failure to comply and/or to accurately and completely report the potential financial conflicts of interest could lead to the following: 1) Prior to publication, article rejection, or 2) Post-publication, sanctions ranging from, but not limited to, issuing a correction, reporting the inaccurate information to the authors' institution, banning authors from submitting work to ASN journals for varying lengths of time, and/or retraction of the published work.

Name: Brian Rayner
Manuscript ID: CJASN-2025-001670R1
Manuscript Title: Kidney and Survival Outcomes with Semaglutide by Chronic Kidney Disease Severity in the FLOW Trial
Date of Completion: January 7, 2026
Disclosure Updated Date: January 7, 2026

ASN
American Society of Nephrology
JASN
Journal of the American Society of Nephrology
KIDNEY360
Accessing Our World From Every Angle
CJASN
Clinical Journal of the American Society of Nephrology

ASN Journal Disclosure Form

As per ASN journal policy, I have disclosed any financial relationships or commitments I have held in the past 36 months as included below. I have listed my Current Employer below to indicate there is a relationship requiring disclosure. If no relationship exists, my Current Employer is not listed.

P. Rossing reports the following:
Employer: Steno Diabetes Center Copenhagen; Research Funding: Novo Nordisk , AstraZeneca, Bayer, Lexicon Pharma; Honoraria: Boehringer Ingelheim, AstraZeneca, Abbott, Novo Nordisk, all honoraria to institution; and Advisory or Leadership Role: Astra Zeneca Bayer , Novo Nordisk, Gilead all honoraria to institution.

I understand that the information above will be published within the journal article, if accepted, and that failure to comply and/or to accurately and completely report the potential financial conflicts of interest could lead to the following: 1) Prior to publication, article rejection, or 2) Post-publication, sanctions ranging from, but not limited to, issuing a correction, reporting the inaccurate information to the authors' institution, banning authors from submitting work to ASN journals for varying lengths of time, and/or retraction of the published work.

Name: Peter Rossing
Manuscript ID: CJASN-2025-001670R1
Manuscript Title: Kidney and Survival Outcomes with Semaglutide by Chronic Kidney Disease Severity in the FLOW Trial
Date of Completion: December 1, 2025
Disclosure Updated Date: March 19, 2025

ASN American Society of Nephrology | JASN Journal of the American Society of Nephrology | KIDNEY360 Accessing Our World From Every Angle | CJASN Clinical Journal of the American Society of Nephrology

ASN Journal Disclosure Form

As per ASN journal policy, I have disclosed any financial relationships or commitments I have held in the past 36 months as included below. I have listed my Current Employer below to indicate there is a relationship requiring disclosure. If no relationship exists, my Current Employer is not listed.

K. Tuttle reports the following:
Employer: Providence Medical Research Center/Providence Inland Northwest Health; Consultancy: Boehringer Ingelheim, Novo Nordisk, Bayer, Lilly, ProKidney, Astra Zeneca, Alnylam, Roche, Glaxo Smith Kline; Research Funding: Travere, Otsuka; Honoraria: Novo Nordisk, Boehringer Ingelheim, Bayer, Travere; Patents or Royalties: Up-to-Date; Advisory or Leadership Role: Chair, Diabetic Kidney Disease Collborative, American Society of Nephrology (unpaid); Chair, 2025 Kidney Week program committee, American Society of Nephrology (unpaid);; and Speakers Bureau: Novo Nordisk.

I understand that the information above will be published within the journal article, if accepted, and that failure to comply and/or to accurately and completely report the potential financial conflicts of interest could lead to the following: 1) Prior to publication, article rejection, or 2) Post-publication, sanctions ranging from, but not limited to, issuing a correction, reporting the inaccurate information to the authors' institution, banning authors from submitting work to ASN journals for varying lengths of time, and/or retraction of the published work.

Name: Katherine R. Tuttle
Manuscript ID: CJASN-2025-001670R1
Manuscript Title: Kidney and Survival Outcomes with Semaglutide by Chronic Kidney Disease Severity in the FLOW Trial
Date of Completion: January 7, 2026
Disclosure Updated Date: January 7, 2026
